# Synthetic Membrane Shaper for Controlled Liposome Deformation

**DOI:** 10.1021/acsnano.2c06125

**Published:** 2022-11-28

**Authors:** Nicola De Franceschi, Weria Pezeshkian, Alessio Fragasso, Bart M. H. Bruininks, Sean Tsai, Siewert J. Marrink, Cees Dekker

**Affiliations:** † Department of Bionanoscience, Kavli Institute of Nanoscience Delft, Delft University of Technology, 2629 HZDelft, The Netherlands; ‡ Groningen Biomolecular Sciences and Biotechnology Institute and Zernike Institute for Advanced Materials, University of Groningen, Nijenborgh 7, 9747 AGGroningen, The Netherlands; § The Niels Bohr International Academy, Niels Bohr Institute, University of Copenhagen, 17DK-2100Copenhagen, Denmark

**Keywords:** membrane deformation, cell division, stomatocyte, dumbbell, FtsZ, Dynamin A

## Abstract

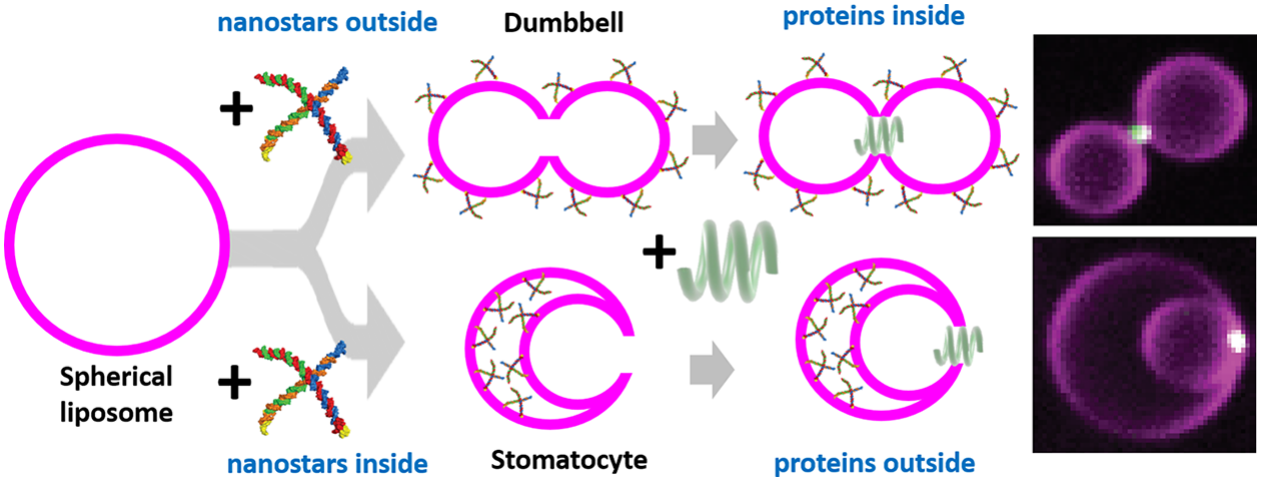

Shape defines the structure and function of cellular membranes. In cell division, the cell membrane deforms into a “dumbbell” shape, while organelles such as the autophagosome exhibit “stomatocyte” shapes. Bottom-up in vitro reconstitution of protein machineries that stabilize or resolve the membrane necks in such deformed liposome structures is of considerable interest to characterize their function. Here we develop a DNA-nanotechnology-based approach that we call the synthetic membrane shaper (SMS), where cholesterol-linked DNA structures attach to the liposome membrane to reproducibly generate high yields of stomatocytes and dumbbells. In silico simulations confirm the shape-stabilizing role of the SMS. We show that the SMS is fully compatible with protein reconstitution by assembling bacterial divisome proteins (DynaminA, FtsZ:ZipA) at the catenoidal neck of these membrane structures. The SMS approach provides a general tool for studying protein binding to complex membrane geometries that will greatly benefit synthetic cell research.

Biological membranes constitute the chassis of cells in all living organisms, providing structural support, compartmentalization, and a platform for organizing biochemical reactions. A fascinating and essential property of membranes is their ability to adopt a variety of shapes, a feature that is exemplified in the rich repertoire of morphologies observed in intracellular organelles.^1^ Two shapes constitute particularly important membrane geometries in cells (Figures 1a and S1A): The dumbbell shape mimics the geometry of a dividing cell, while the stomatocyte shape recapitulates the membrane topology found in several intracellular organelles including the nuclear envelope and the open autophagosome. A common feature that is shared by both dumbbells and stomatocytes is a neck, a double-membrane pore with the geometrical shape of a catenoid that features both positive and negative membrane curvature (Figure 1a). Membrane deformations result from the combined effects of the spontaneous and induced local curvatures.^2^ The molecular origin of spontaneous curvature can be explained by a number of mechanisms. It can, for example, result from asymmetries in lipid composition in the bilayer, from bulky groups that insert into one leaflet of the membrane (“wedges”), from oligomerization of membrane proteins that build up complexes with an intrinsic curvature (“scaffolding”), and from molecular crowding due to entropic repulsion of soluble protein domains arising from confinement in a crowded environment. From previous studies it appears that scaffolding and wedging are the most effective ways to induce curvature, with crowding having a more modest effect.^3,4^

**Figure 1:**
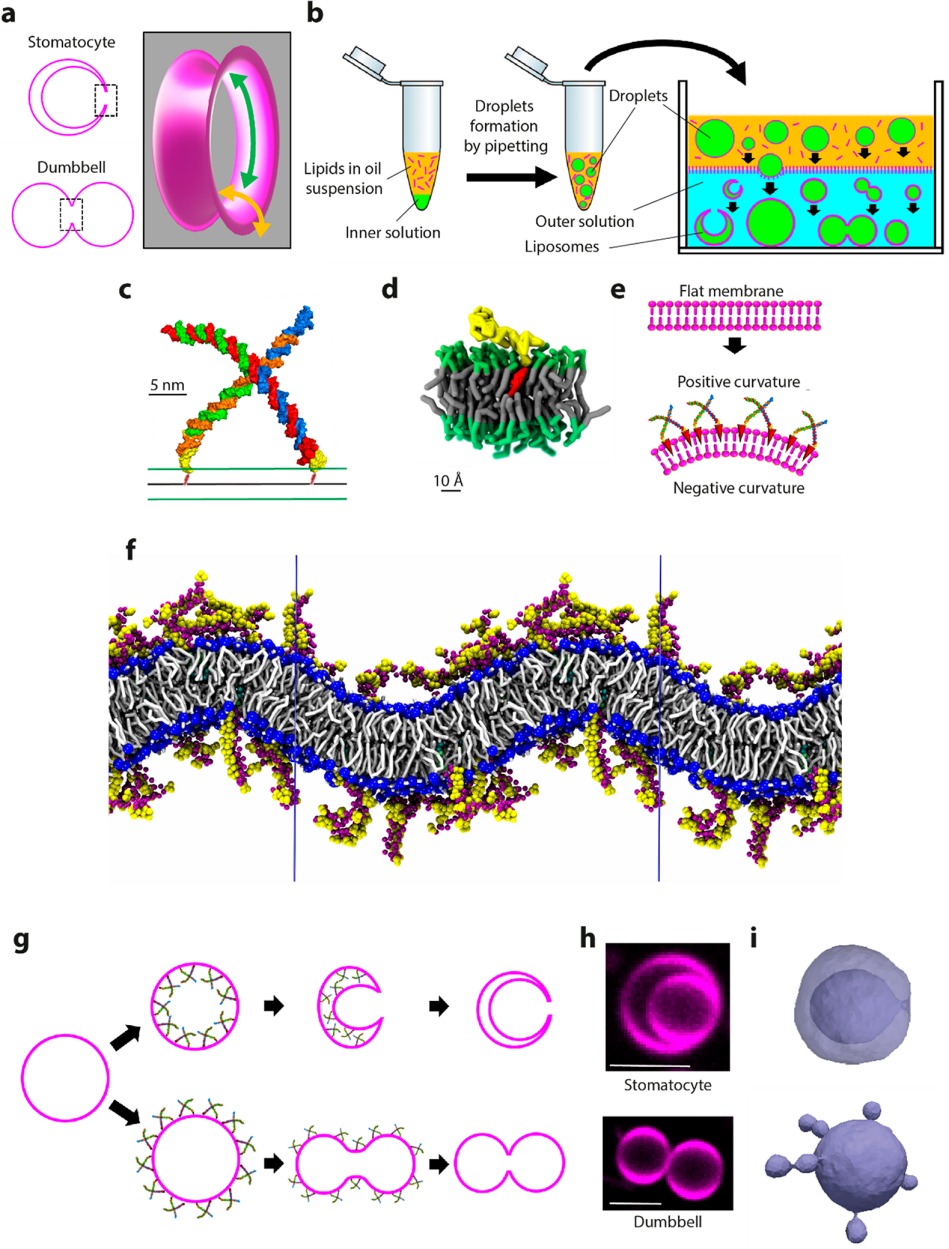
Membrane shaping by the SMS approach. (a) Schematics showing the toroidal geometry of a double-membrane pore (right) present in both stomatocyte (top) and dumbbell shapes (bottom). Negative and positive membrane curvatures are indicated by the green and orange arrows, respectively. (b) Schematics depicting liposome production with the SMS approach. Water-in-oil droplets are produced by pipetting and then deposited in the observation chamber, where they sink by gravity. The bilayer is created when the droplets cross the interface between oil and aqueous (outer) solution. An osmotic pressure difference between inner and outer solutions induces shape deformations in the newly formed liposomes. Inner solution is denoted in green, oil phase in orange, lipids in magenta, and outer solution in cyan. (c) Structural model of a single nanostar formed by four ssDNA oligos (red, blue, orange, green) and hybridized to the chol-oligos (yellow). The position of the cholesterol moiety (red) embedded within the membrane bilayer (green and black lines) is schematically indicated. (d) Representative snapshot of an MD simulation of a chol-oligo inserted into the membrane (color-coding as in panel c). (e) Schematic illustrating curvature generation by the combination of nanostars and chol-oligo. (f) Snapshot of a bilayer containing 10 chol-oligo molecules in each monolayer. The projected area of the bilayer is fixed helping the membrane to bend. The simulation box is shown as a blue frame. (g) Shape transformations resulting from assembling the nanostars on either the inner or the outer side of liposomes. (h) Example of a confocal image of stomatocyte and dumbbell shaped liposomes generated by the SMS. Scale bars: 5 μm. (i) Stomatocyte and dumbbell obtained by DTS simulations.

An elegant body of knowledge has been developed with theoretical calculations describing membrane shape transformation of spherical liposomes into a wide variety of shapes as a result of varying the membrane spontaneous curvature and surface-to-volume ratio.^5−7^ Experimentally, membrane deformation has been extensively studied in osmotically deflated liposomes.^2^ Curvature can be induced by a wide range of membrane-binding molecules^8,9^ including proteins,^10,11^ large DNA molecules^12^ and DNA assemblies,^13,14^ sugars^15^ and dextran^16^ as well as induced by Min protein activity^17^ or active particles.^18^ Stomatocyte-like shapes have, however, rarely been reported in literature—merely as short-lived intermediates in “vesicles-in-vesicles” membrane systems.^19^ The ability to reproduce these shapes in vitro for quantitative characterization of the assembly and function of protein complexes on their neck regions would be beneficial for bottom-up synthetic biology. In dumbbells, for example, the neck represents the assembly site for the division machinery, while in stomatocytes, the neck is the assembly site of the nuclear pore complex (NPC) at the nuclear envelope as well as the membrane topology where the ESCRT-III complex binds. Due to the unavailability of good model systems, only sporadic examples of such protein reconstitutions are available.^20^ A common drawback of the aforementioned experimental approaches for producing dumbbells and stomatocytes is the requirement for specific lipid and buffer compositions that restrict their applicability to co-reconstitute proteins in order to study biological processes. Furthermore, comparison between different membrane geometries is hardly possible since each system is designed to obtain one specific shape, and poor control and low yields limit quantitative characterization of complex membrane shapes.

Here, we establish an approach that we name the “synthetic membrane shaper” (SMS) to shape liposomes in a highly controlled way. The SMS is a method in which small DNA nanostructures adsorb onto the surface of a liposome that is being formed in a hyperosmotic environment. This induces and stabilizes large-scale membrane deformations, generating both dumbbell and stomatocyte shapes. Importantly, the SMS works within a broad range of membrane and buffer compositions and this makes it widely applicable for protein reconstitution, as we show by assembling various proteins on high-curvature regions such as necks. We use coarse-grain molecular dynamics (MD) simulations^21,22^ to explain the curvature inducing effect of the DNA nanostructures, as well as dynamically triangulated surfaces (DTS) simulations^23,24^ to verify the vesicle shape transformations and characterize the assembly of proteins to regions of complex membrane curvature.

## Results

### Establishing a Synthetic Membrane Shaper to Morph Liposomes into Dumbbells or Stomatocytes

Liposomes were generated in a gentle method where water-in-oil droplets slowly crossed an oil/water interface by gravity (Figure 1b). This method was chosen to overcome harsh procedures in many common production and handling techniques for liposomes that, due to centrifugation and pipetting, can cause loss of delicate membrane structures. Droplets were prepared by pipetting the inner aqueous solution into a lipid-in-oil dispersion, whereby water-in-oil droplets with a lipid monolayer were formed. Subsequently, these droplets were transferred by gravity through an oil/water interface to acquire a second bilayer leaflet and thus form liposomes in a hyperosmotic outer solution in an observation chamber, where liposomes settled at the bottom where they were imaged with fluorescence microscopy. With this liposome-production technique, it is very easy to include macromolecules such as proteins or DNA in both the inner and outer solutions, while preserving fragile membrane structures. Membrane shape transformation can be driven by the osmotic difference between the inner and outer solutions, regulating the surface-to-volume ratio. We found that an osmotic difference of 20–40 mOsm was suitable for producing deformed liposomes in our system. This resulted in membrane deformations that however were not greatly controlled, with both the total yield and the relative occurrence of different shapes varying greatly across preparations. A variety of morphologies was observed, including dumbbell and stomatocyte shapes, often coexisting in the same preparation (Figure S1B).

In order to increase the yield and reproducibility of structures, we developed an approach to drive vesicles into a defined shape. We used DNA nanostars,^25^ which are 96.5 kDa tetrameric cross-shape DNA assemblies (Figure 1c) that at two positions have a binding site to a short complementary oligonucleotide (chol-oligo) that is functionalized with a cholesterol moiety at its 3′ end. This combination of the action of the DNA nanostars/chol-oligo complex with the gentle liposome production method constitutes the SMS approach. As we detail below, this SMS approach was found to enable the production of two kinds of membrane shapes: stomatocyte-shaped liposomes when the nanostars were bound to the inner leaflet, and dumbbells for binding of the nanostars to the outer leaflet (Figure 1g,h).

From a theoretical point of view, the range of the osmolarity (mM) used in our experiment imposes a strong constraint on the vesicle volume. Meaning, that the bending energy of transforming a spherical vesicle into dumbbell or stomatocyte shapes is much smaller than the energy required to make a significant change in the vesicle volume (see DTS method section). Therefore, the osmolarity difference between in and out determines the vesicle volume. DTS simulations show that only a limited number of shape classes can be formed in the absence of any induced spontaneous curvature. For instance, a stomatocyte shape can be generated when the vesicle volume is reduced to 50% of a spherical vesicle (Figure S2), consistent with previous findings.^5^ However, upon applying negative and positive membrane curvature (here induced by the nanostars/chol-oligo complex bound to inner or outer membrane, respectively) stomatocyte and dumbbell structures in a much wider range of reduced volumes (Figures 1i and S2 and Movies 1 and 2). Very large values of the spontaneous curvature furthermore caused the appearance of multispherical shapes (Figure S2), in line with previous studies.^26^ These results indicate that the spontaneous membrane curvature is an essential parameter for generating a diverse range of membrane shapes.

We hypothesize that insertion of the chol-oligo into the membrane generates curvature by the wedge effect, while the nanostars induce a further bilayer asymmetry by molecular crowding. MD simulations of the chol-oligo in model membranes indicated that the DNA strand, being covalently attached to cholesterol, is partially dipping into the polar headgroup region of the bilayer (Figure 1d). As it is detailed in the Supporting Information (Figures S3–S7, Table S1, and Supplementary Note 1), the partial insertion of the DNA strand causes expansion of the distance between neighboring lipids, indeed generating membrane curvature through wedging. It is worth noticing that since only few initial (DNA) bases enter the polar region, the length of the oligo chain does not contribute significantly to these structural changes, consistent with the experimental results (see below). Based on the MD results, we estimate that a substantial curvature is induced by a single chol-oligo, ∼ 0.3 nm^–1^ (Supplementary Note 1). Therefore, even a low concentration of bound chol-oligos should already lead to large membrane deformations. The ability of chol-oligo to induce positive curvature (i.e., curving the membrane in the direction away from the bound leaflet, Figure 1e) is further evidenced by MD simulations of buckled membrane patches, showing preferential binding of the constructs to regions of high positive membrane curvature (Figure 1f).

### Characterization of Stomatocytes and Dumbbells Produced with the SMS

**Figure 2:**
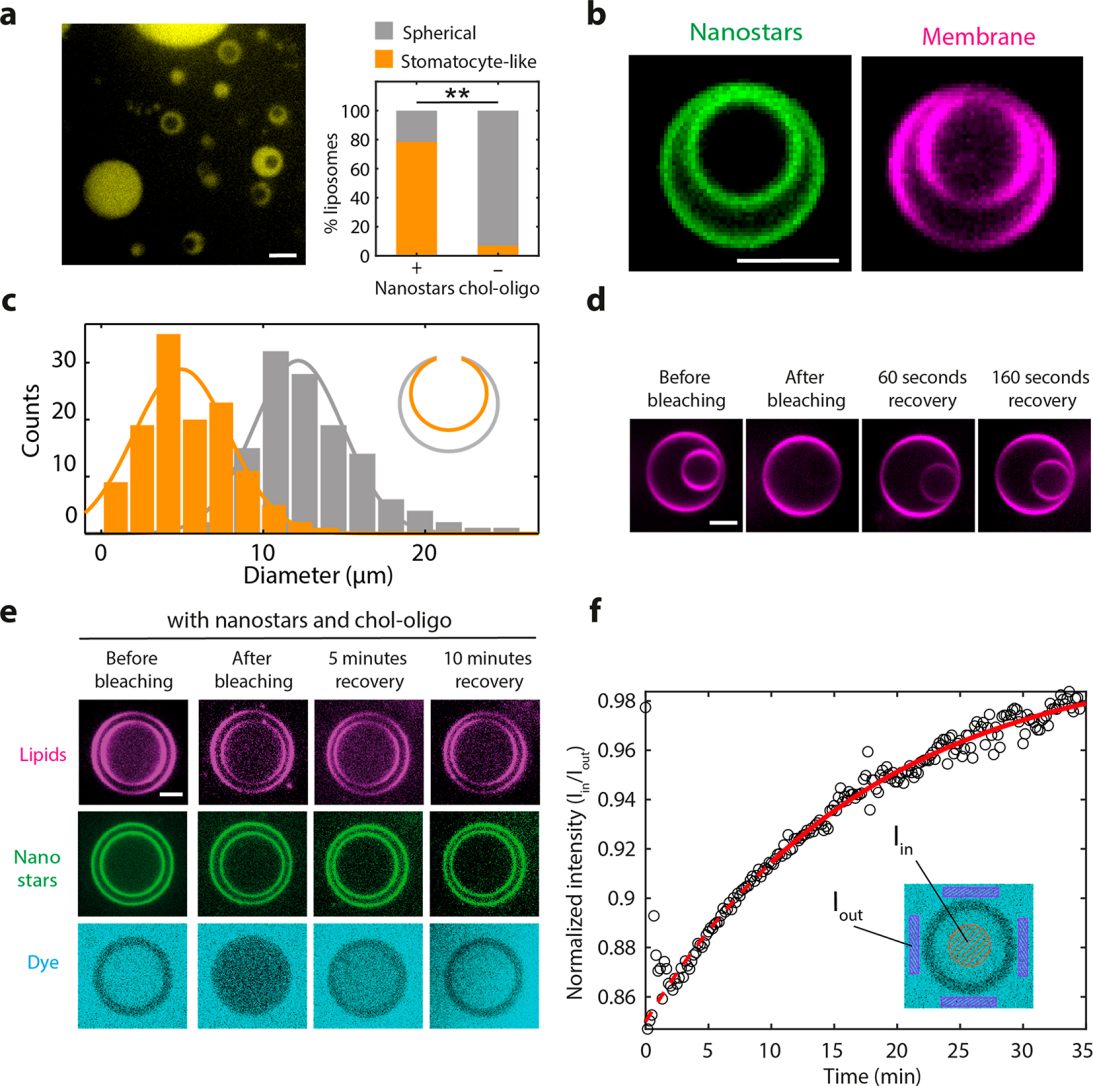
Characterization of stomatocytes produced with SMS. (a) Example of a field of view in a SMS stomatocyte preparation. A soluble dye (visible in yellow) was encapsulated in the lumen of the stomatocytes. The plot (right) shows the quantification of the number stomatocytes obtained with or without nanostars/chol-oligo (*N* = 511 liposomes from 3 independent preparations). (b) Representative example of a stomatocyte, imaged both in the lipid (magenta) and nanostars (green) fluorescence. Nanostars were observed to be uniformly distributed over the membrane. (c) Distribution of the diameter of the inner and outer membrane of stomatocytes. Outer diameter was 13.1 ± 3.7 μm (average ± SD); inner diameter was 5.4 ± 2.7 μm (average ± SD). *N* = 125 stomatocytes from 5 independent preparations. (d) FRAP experiment on fluorescent lipids. The inner membrane is bleached, and fluorescent recovery over time is visualized. (e) FRAP experiment of soluble Alexa-647 dye contained in the lumen of the stomatocyte obtained by the SMS approach in the presence of nanostars/chol-oligo. Stomatocytes were produced in the presence of Alexa-647. The dye present in the inner compartment is photobleached and its recovery through the toroidal pore is visualized over time. (f) Plot showing the normalized intensity of the dye in function of time. The inset depicts the regions where *I*_in_ (fluorescence intensity in the inner compartment, in red) and *I*_out_ (fluorescence intensity in the outer buffer, in purple) were measured. Solid line denotes a fit of eq 7 (Supplementary Note 2). All scale bars: 5 μm.

The presence of chol-oligo/nanostars in the inner aqueous solution of the liposomes strongly increased the yield of stomatocytes, i.e., from 7% without to 78% with nanostars (Figure 2a). Nanostars/chol-oligo were found to be distributed homogeneously throughout the membrane surface without clustering at the microscale (Figure 2b). MD simulations indicated that the chol-oligos also did not form stable clusters at the nanoscale (Figure S8). The SMS approach to induce shape deformations appeared to work efficiently on liposomes with a size in the lower μm range (Figure 2c). Such range is perfectly suited for in vitro reconstitution because it allows to clearly recognize these membrane structures by optical microscopy while at the same time being similar to the size of eukaryotic cells. We also separately investigated the contributions of the chol-oligos and nanostars in mediating membrane deformation. Our data suggest that the insertion of chol-oligo into the membrane (i.e., wedging) is sufficient to induce efficient inward budding, and that the length and sequence of the chol-oligo is not relevant (Figure S9A).

By optical imaging alone it cannot be determined whether the neck connecting the inner and outer membrane in stomatocytes is open. Indeed, while in few instances an elongated neck could be imaged (Movie 3), the vast majority of stomatocytes showed a neck that was very short and localized between the adjacent inner and outer membranes. We therefore performed a series of FRAP (fluorescence recovery after photobleaching) experiments to validate the presence of an open neck. After photobleaching the fluorescent lipids of the inner vesicle, we visualized the fluorescence recovery and equilibration of lipids between inner and outer vesicle (Figure 2d). Furthermore, we generated stomatocytes in the presence of a soluble dye in the outer solution. As expected, the dye was observed in both the inner vesicle and the exterior of the stomatocytes right after production. We then diluted the external solution with buffer that did not contain dye, thereby testing whether the dye would exit the inner vesicle through an open pore, or would be retained in case the pore was closed. In this way, we estimated that the majority of stomatocytes (55%) clearly exhibited an open neck (Figure S9B).

FRAP experiments also allowed to estimate the neck size. Figure 2e shows a characteristic example of a FRAP recovery in a stomatocyte, where first the inner vesicle was photobleached, followed by recovery of the small soluble dye (Alexa488) within tens of minutes. By fitting a diffusion model (as described in ref (27); Supplementary Note 2 and Figure S10) to the data (Figure 2f), we could estimate an open pore diameter of 134 ± 103 nm (*N* = 9, error is SD). This is significantly smaller compared to the control experiment on stomatocytes that were formed in absence of nanostars and chol-oligo, where recovery occurred within tens of seconds, corresponding to a pore size of 1.3 ± 1.2 μm (N = 8, error SD; see Figure S11). The overall stomatocyte shape corroborated this estimation: while stomatocytes generated with chol-oligo/nanostars were invariably spherical (Figure 2b,d,e) and did not display membrane fluctuations, those obtained in the absence of chol-oligo/nanostars were often nonspherical (Figure S12), indicating a low level of membrane tension. In tube-pulling experiments, increasing the membrane tension resulted in steeper neck curvature.^28^ When chol-oligo/nanostars were present, the stomatocytes appeared more tense, and accordingly, they featured a narrower and more curved neck region. The necks generated using the SMS are close to the range of neck sizes found in cells: for instance, the assembly site of the NPC at the nuclear envelope is 120 nm wide,^29^ while the assembly site for the ESCRT complex in cytokinesis can range between several hundreds of nm until the point of scission.^30^

**Figure 3:**
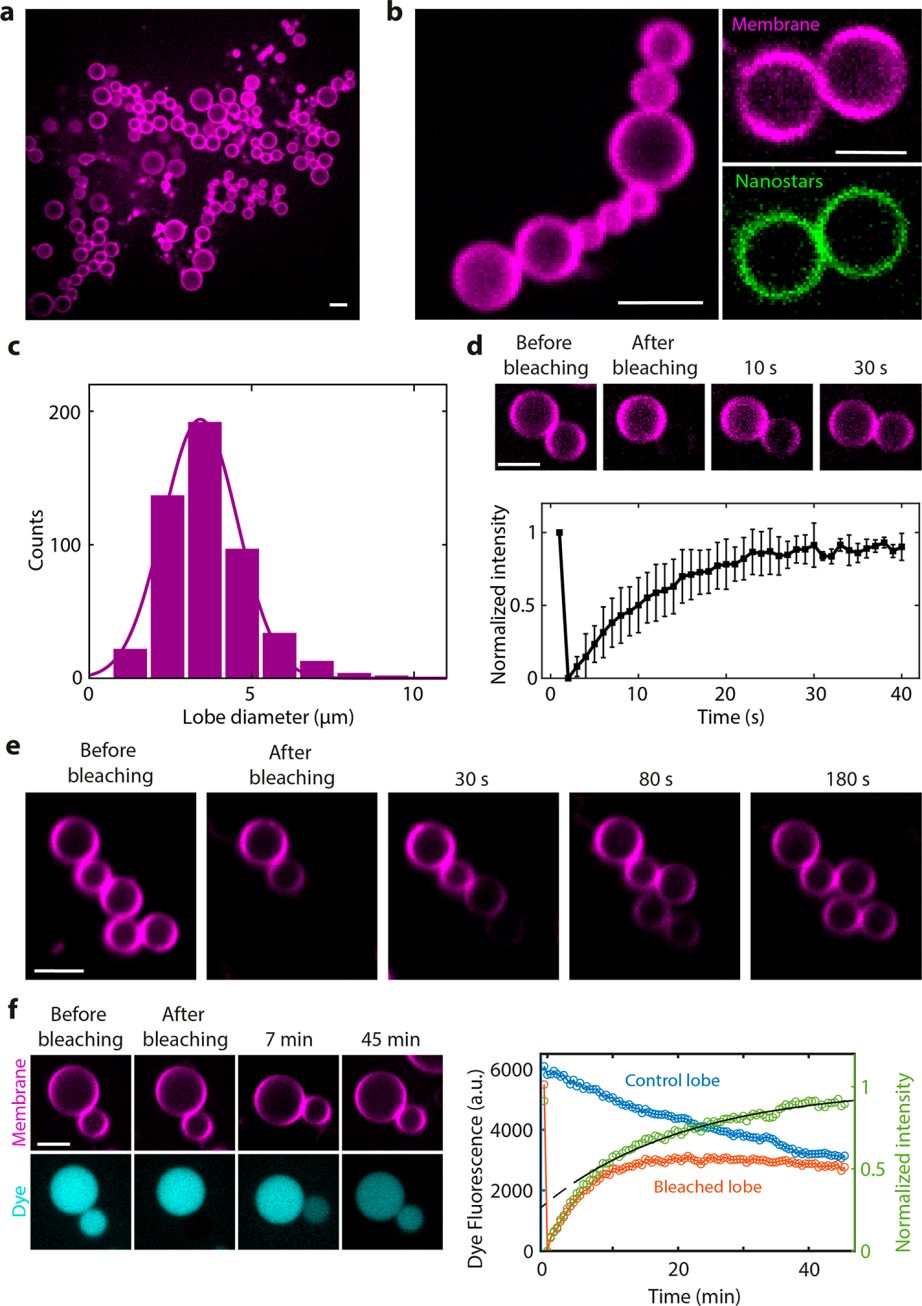
Characterization of dumbbells produced with SMS. (a) Large field of view in a SMS dumbbell preparation. (b) Example of a chain of dumbbells (left) and a dumbbell (right). The nanostars (green) are observed to be homogeneously distributed throughout the membrane. (c) Quantification of dumbbell lobe diameter. *N* = 501 lobes from 6 independent preparations. (d) Recovery of fluorescent lipids upon FRAP photobleaching of one of the two lobes of dumbbells. The plot shows the average recovery time of the fraction of dumbbells (81%) that exhibited full recovery. *N* = 31 dumbbells from 6 independent preparations. (e) FRAP recovery of fluorescent lipids upon photobleaching of part of a chain of dumbbells. (f) FRAP experiment showing flowing of soluble dye between lobes of a dumbbell. The right plot shows the fluorescent intensity versus time. Solid line denotes a fit of eq 16 (Supplementary Note 3). All scale bars: 5 μm.

Liposome SMS preparations where nanostars and chol-oligo were added on the *outside* at 250 nM and 500 nM, respectively, were found to be enriched in dumbbells and chains of dumbbells (Figure 3a,b). Higher concentration of nanostars and chol-oligo (1 μM) resulted in small lobes connected by membrane nanotubes. This was likely due to curvature generation induced by the nanostars and chol-oligo. As observed for the stomatocytes, the diameter of the lobes in these dumbbells were limited to the low μm range (Figure 3c), and the dumbbells were tenser compared to analogous structures obtained without chol-oligo/nanostars (Figure S13 and Movies 4 and 5). In the majority (81%) of dumbbells observed, FRAP analysis indicated that lipids did flow across the neck, confirming that the lipid membranes of adjacent lobes were connected (Figure 3d and Movie 6). Accordingly, when performing FRAP experiments on chains of dumbbells, we observed fluorescent-lipid recovery that proceeded sequentially from each lobe to the next (Figure 3e). Upon encapsulation of soluble dye within the lumen of these dumbbells and photobleaching of one lobe, we observed dye recovery (Figure 3f), indicating that the lumens of adjacent lobes are in mutual communication via an open neck. Figure 3f shows fitting of the FRAP recovery data to a diffusion model that describes the molecular flow in a dumbbell two-vesicle system (see Supplementary Note 3 and Figure S14), from which we estimate an open pore diameter of 26 ± 23 nm (*N* = 7, error is SD). The data indicate that the large majority of the structures that we obtained were indeed true dumbbells rather than individual liposomes that were adhering to each other post hoc. While sporadic and qualitative examples of lipids FRAP assay in dumbbells have been reported,^11^ there were so far, to the best of our knowledge, no quantitative FRAP experiments of lipids and encapsulated dyes for dumbbells and stomatocytes, including the theoretical analysis framework and estimated pore size.

### Dynamin A and FtsZ:ZipA Proteins Assemble at the Necks of Stomatocytes and Dumbbells

**Figure 4:**
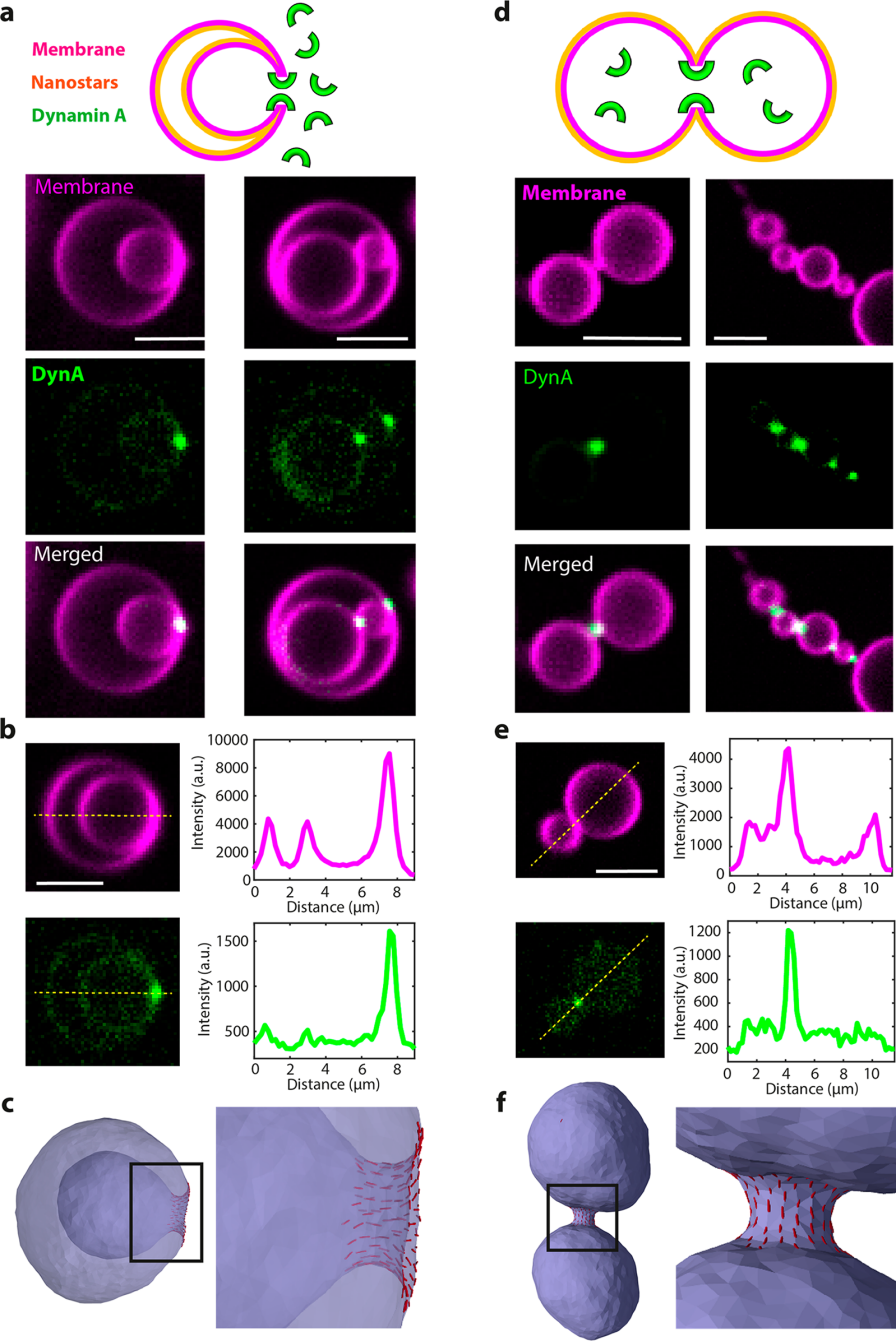
Assembly of Dynamin A on stomatocyte- and dumbbell-shaped liposomes. (a) Assembly of a Dynamin A on stomatocytes generated by SMS. Schematic on the top row clarifies the topology of the protein and the nanostars with respect to the membrane. Lower panels show representative images from 3 independent preparations. (b) Line scan analysis of Dynamin A enrichment at necks of stomatocytes. (c) DTS simulation of a vesicle under osmotic pressure and negative global curvature and 5% protein bound on the outside. Each red line represents one protein and its orientation on the membrane. Proteins inducing positive membrane curvature enrich in the neck region. See also Figure S19. (d) Assembly of Dynamin A inside dumbbells generated by SMS. Schematic on the top row clarifies the topology of the protein and the nanostars with respect to the membrane. Lower panels show representative images from 3 independent preparations. (e) Line scan analysis of Dynamin A enrichment at necks of dumbbells. (f) DTS simulation of a vesicle under osmotic pressure and positive global curvature and 5% protein coverage. The proteins, which are bound to the membrane from the inside, induce positive membrane curvature and enrich in the neck region. See also Figure S20. All scale bars 5 μm.

Subsequently, we used the SMS approach to characterize protein assembly at necks of dumbbells and stomatocytes. We first tested the assembly of Dynamin A (DynA). This bacterial member of the dynamin superfamily localizes to the neck of dividing cells where it has been proposed to be involved in the final step of membrane scission.^31^ However, thus far DynA could never be reconstituted and imaged in vitro on membrane necks. We reconstituted DynA in both stomatocytes and dumbbells obtained by SMS. DynA binding to the membrane was obtained by incorporating 5% mol/mol of negatively charged DOPG in the lipid mix (Table S6). Note that in both cases, the protein and the nanostars are bound to opposite sides of the membrane (see schematics in Figure 4a,d) and hence they will not directly interact.

We found DynA to be highly enriched at membrane necks of both stomatocytes and dumbbells (see Figure 4a,d and Movie 7. This enrichment directly indicates that DynA is able to sense membrane curvature. Protein enrichment on membrane necks and membrane nanotubes can be quantified by the sorting ratio *S*_R_,^32^ a dimensionless parameter that indicates the fluorescence intensity of the protein at the neck normalized to that of protein bound on the liposome outer surface (see Methods). A sorting ratio of *S*_R_ > 1 indicates enrichment at the neck. For DynA on stomatocytes, we measured a *S*_R_ of 8.3 ± 4.8 (*N* = 28; error is SD; Figure 4b). On dumbbells, the *S*_R_ was even higher, but it could not be reliably quantified because of the extremely low binding to the liposome surface (Figure 4e).

DynA reconstitution highlights the advantages of using the SMS approach over existing methodologies. A widely used approach in the field is to encapsulate proteins using inverted emulsion^33^ and subsequently raising the osmolarity of the outer solution. With this protocol, we did observe membrane shape transformation; however, the few DynA clusters that were present almost invariably localized away from membrane necks (Figure S15A). When we encapsulated DynA using the SMS approach but without adding chol-oligo/nanostars, we observed extensive protein and lipid aggregation, with very few dumbbells present and no DynA clusters at necks (Figure S15B). This is likely due to the fact that DynA binds to lipids during the process of liposome formation and interferes with this process. Only by reconstituting DynA with the SMS approach including chol-oligo/nanostars we obtained a high yield of clean dumbbells and chains of dumbbells where most of the DynA clusters localized at necks (Figure S15C).

**Figure 5:**
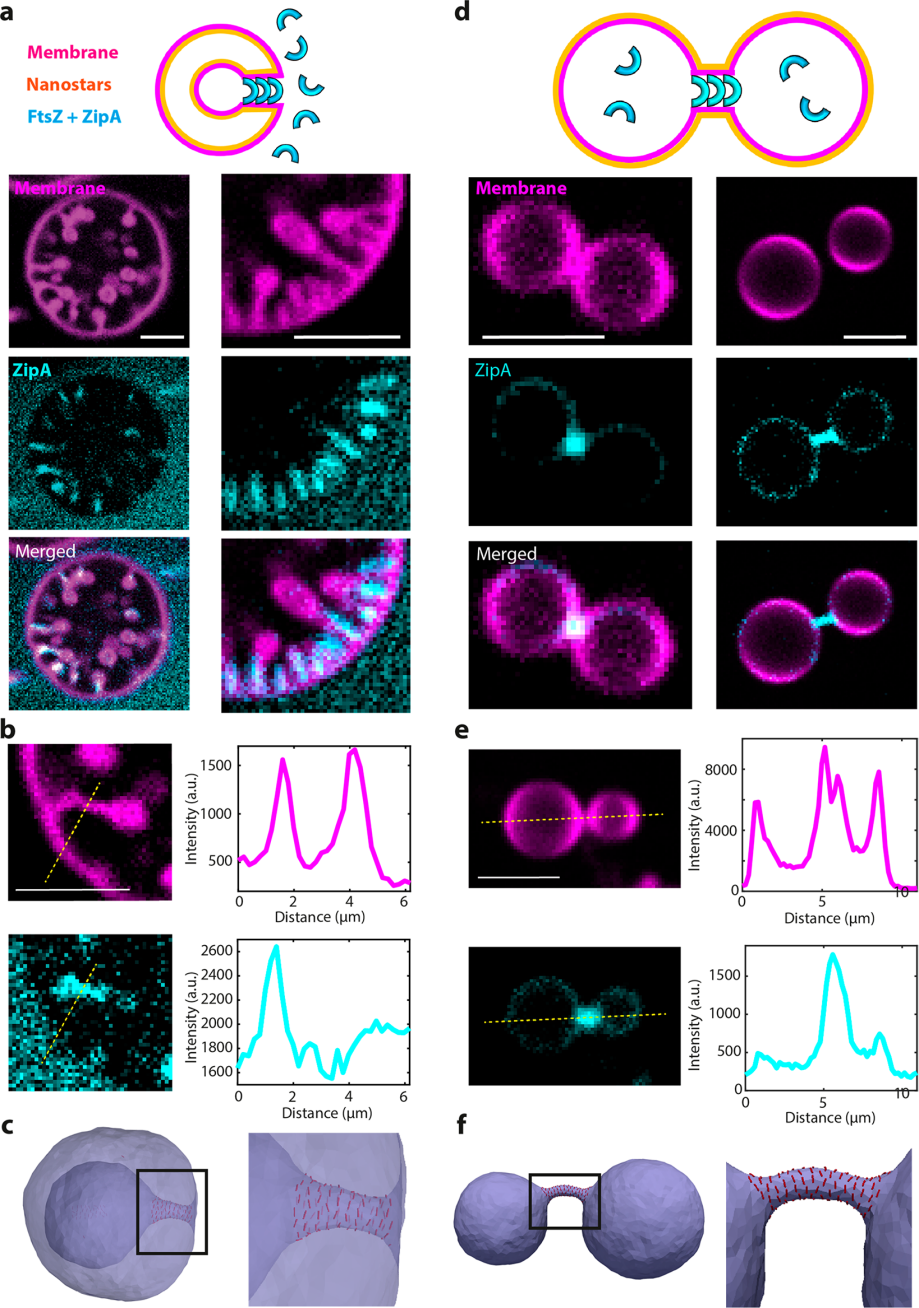
Assembly of FtsZ:ZipA on stomatocyte- and dumbbell-shaped liposomes. (a) Assembly of FtsZ:ZipA:GTP on stomatocytes generated with SMS. Schematic on the top row clarifies the topology of the protein and the nanostars with respect to the membrane. Lower panels show representative images. (b) Line scan analysis of FtsZ:ZipA enrichment at necks of stomatocytes. (c) DTS performed for a fixed reduced volume and 2% protein coverage on the outside membrane show that proteins inducing negative curvature localize at the neck region. Each red line represents one protein and its orientation on the membrane. Similar behavior was obtained when the volume was reduced by applying osmotic pressure difference (see Figure S19). (d) Assembly of FtsZ:ZipA:GTP inside dumbbells generated with SMS. Schematic on the top row clarifies the topology of the protein and the nanostars with respect to the membrane. Lower panels show representative images from 3 independent preparations. (e) Line scan analysis of FtsZ:ZipA enrichment at necks of dumbbells. (f) DTS simulation of a vesicle under osmotic pressure, positive global curvature, and 5% protein coverage on the inside membrane. Proteins inducing negative curvature localize at the neck region. See also Figure S20. All scale bars 5 μm.

We also tested the assembly of FtsZ, the major structural component of the bacterial Z-ring that has been extensively studied before.^34^ In vitro, FtsZ is known to assemble on negatively curved membranes.^35^Figure 5a and Movie 8 illustrate the assembly of a FtsZ, along with its membrane anchor ZipA,^36^ on stomatocytes that were formed by SMS. Ftsz/ZipA binding to the membrane was obtained via the Histidine tag present on ZipA by incorporating 2% mol/mol of DGS-NTA(Ni) in the lipid mix (Table S6). Interestingly, rather than a single internal compartment, the FtsZ proteins induce the formation of an extensive array of inward elongated necks that often end with a small spherical internal compartment. The elongated necks indicate that the FtsZ:ZipA complex is able to actively generate negative curvature (see below), as similarly reported for other proteins.^37^ This appears to affect the very process of stomatocyte formation, where likely the process of forming one large internal compartment is stalled by the FtsZ as it stabilizes a narrow neck, and subsequently many invaginations occur, leading to an array of necks. The average *S*_R_ for the FtsZ:ZipA complex was found to be 4.0 ± 1.8 (*N* = 45; error is SD; Figure 5b). When FtsZ was assembled inside dumbbells, elongated necks were also often observed experimentally (Figure 5d and Movie 9), confirming that FtsZ:ZipA complex can generate negative curvature. In dumbbells, we measured a higher average *S*_R_ of 13.2 ± 9.2 (*N* = 30; error is SD; Figure 5e). Taken together, the data indicate that FtsZ:ZipA complexes assemble preferentially on negatively curved membranes as well as are able to actively generate negative membrane curvature.

In line with the above experiments, we performed DTS simulations on the obtained dumbbell or stomatocyte structures and added different concentrations of proteins, testing different settings for protein–membrane and protein–protein interactions (Figures S16–S20). The results show that, in absence of protein–protein interactions, no significant enrichment of proteins in the neck was observed (Figure S18). Including protein–protein interactions, however, the proteins do accumulate at the neck regions in agreement with the experimental results (Figures 4c,f and 5c,f and Figures S19 and S20), suggesting that FtsZ:ZipA complexes and DynA possibly directly interact or form filaments on the membrane surface. Last but not least, our results show that proteins that induce a negative membrane curvature are capable of elongating the neck and adjust the neck to a specific size, while proteins that induce positive membrane curvature fail to elongate the neck (Figure S17). Matching these results with the experimental data, we find that FtsZ:ZipA induces a negative membrane curvature while DynA induces positive membrane curvature (Figures 4c,f and 5c,f).

## Conclusions

We presented an approach called SMS to induce large-scale membrane deformation in liposomes, which allows to obtain high yields of stomatocyte or dumbbell shapes. Photobleaching experiments confirmed the membrane topology of these structures and allowed to estimate the 30–130 nm size of their defining feature, the catenoid-shaped pore at the neck. Next to developing SMS, we demonstrated that it provides an excellent platform to study the interaction of bacterial proteins with curved membranes at the neck region. We found that Dynamin A is able to sense membrane curvature, leading to strong enrichment at the neck of both stomatocytes and dumbbells. As DynA has been suggested to mediate the last step of membrane abscission during bacterial division, encapsulation of DynA in dumbbells by the SMS may serve as a relatively simple experimental setting for reconstituting synthetic cell division by a bottom-up approach. Moreover, we showed that FtsZ:ZipA is able to actively generate negative membrane curvature. Interestingly, this also demonstrates that the membrane structures obtained by the SMS can be further deformed by forces that are exerted by proteins.

Overall, the findings reported here portray the SMS as a promising platform for reconstitution studies of protein binding to membrane necks and in particular on pores that have a catenoid-like shape, which are notoriously difficult to produce with state-of-the-art *in vitro* reconstitution systems. More complex protein complexes that could be reconstituted with this approach include the nuclear pore complex,^38^ the ESCRT-III machinery,^39,32^ and the many proteins involved in cell division in eukaryotes, bacteria, and archaea. Currently, the gold standard in characterization proteins interacting with curved membranes is the tube pulling assay, which allows protein reconstitution on positive curvature^40^ and, by using more complicated procedures, on negative curvature.^28,32,41^ However, tube pulling requires highly specialized equipment and is notoriously challenging to perform. The simple and accessible SMS approach developed here is in many aspects complementary to tube pulling (Table S2) and appears to be particularly suited to study protein assembly on membrane regions with negative curvature. Furthermore, the SMS reproduces the membrane geometry of a toroidal pore, which cannot be achieved by tube pulling, while not requiring any specialized equipment. We anticipate that the SMS will be widely adopted as a general tool for protein and membrane biophysics studies, with good potential to become part of the division machinery for synthetic cells.^42^

## Methods

### Reagents

Glucose (G7021), MgCl_2_ (M8266), silicone oil (317667), mineral oil (M3516-1L), and Optiprep (60% (w/v) iodixanol in water, D1556) were purchased from Sigma-Aldrich. Tris-HCl (10812846001) was purchased from Roche. DOPC (1,2-dioleoyl-*sn*-glycero-3-phosphocholine) (850375), DOPE-PEG(2000) amine (1,2-dioleoyl-*sn*-glycero-3-phosphoethanolamine-*N*-[amino(polyethylene glycol)-2000] (ammonium salt)) (880234), 18:1 (Δ9-Cis) PG (1,2-dioleoyl-*sn*-glycero-3-phospho-(1′-*rac*-glycerol) (sodium salt)) (840475), 18:1 DGS-NTA(Ni) (1,2-dioleoyl-*sn*-glycero-3-[(*N*-(5-amino-1-carboxypentyl)iminodiacetic acid)succinyl] (nickel salt, chloroform) (790404C), and DOPE-Rhodamine (1,2-dioleoyl-*sn*-glycero-3-phosphoethanolamine-*N*-(lissamine rhodamine B sulfonyl) (ammonium salt) (810150C) were purchased from Avanti Lipids. Lipids were stored and resuspended in anhydrous chloroform (288306, Sigma Alrich). Ultrapure bovine serum albumin used for passivation of the glass coverslips was purchased by ThermoFisher. For the influx experiments, we employed 70 kDa dextran-FITC (FITC/glucose (mol/mol) = 0.004) (Sigma-Aldrich) and Alexa Fluor 647 C2 maleimide (A20347, ThermoFisher).

### DNA Constructs

DNA oligos were purchased from IDT. The sequence of the DNA oligos composing the nanostars and chol-oligo are reported in Table S3.

### Buffers and Solutions

Composition of solutions used in SMS preparations are shown in Table S4 (inner solution) and Table S5 (outer solution). We observed that addition of NaCl in the outer buffer promoted strong tubulation and this was therefore avoided.

### Lipid-in-Oil Suspension and Droplet Preparation

Lipid-in-oil suspensions were prepared according to an optimized protocol.^27,43^ Lipids solubilized in chloroform were mixed and blow-dried with nitrogen. Inside a glovebox filled with nitrogen, lipids were subsequently resolubilized with anhydrous chloroform. The freshly prepared mixture of silicone and mineral oil was added to the lipids dropwise while vortexing at 1400 rpm. The lipid-in-oil solution was finally vortexed at 2800 rpm for 2 min and further sonicated in an ice bath for 15 min. The lipids mixes used in this study are described in Table S6. Droplets were generated by manually pipetting inner solution into the lipid-in-oil suspension and subsequently added on top of the outer solution in the observation chamber.

### Data Collection and Analysis

Fluorescence images were acquired at the midplane of liposomes using spinning disk confocal laser microscopy (Olympus IXB1/BX61 microscope, 60× objective, iXon camera) with Andor iQ3 software. To induce photobleaching, we employed raster scanning with a 491 nm laser (at 9.8 mW) over the region of interest. To measure the recovery signal, frames were collected every 1 s, starting right after the photobleaching event. Fluorescence images were analyzed and processed using ImageJ (v2.1.0). The extracted fluorescence data were plotted and fitted using MATLAB.

### Protein Expression, Purification, and Labeling

FtsZ was overexpressed in *Escherichia coli* ER2566 cells (New England Biolabs), purified as described earlier,^44^ and stored in storage buffer (50 mM Tris pH7.5, 500 mM KCl, 5 mM MgCl_2_, 10% glycerol). Labeling of FtsZ with Alexa Fluor 488 NHS ester was performed as described previously.^45^ Purified sZipA was kindly provided by Dr. G. Rivas.^46^*Bacillus subtilis* DynA was overexpressed from pET16b (kindly provided by Dr. M. Bramkamp) and purified essentially as described (Bürmann et al. 2011) but eluted with a linear imidazole gradient instead of a step elution. A solution of ∼10 μM Dynamin was labeled with 8-fold molar excess of Alexa Fluor 488 maleimide in the presence of 0.05 mM TCEP (45 min at room temperature), quenched with 10 mM β-mercaptoethanol, and separated from free label on a Superdex S200 column equilibrated with T5 buffer (50 mM Tris/HCl pH8.0, 500 mM NaCl, 10% glycerol).

### Calculation of the Sorting Ratio

The sorting ratio, *S*_R_, at membrane necks was calculated as in ref (32) using the formula
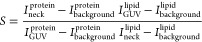
where *I*_neck_^protein^ and *I*_GUV_^protein^ represent the fluorescence intensities of the protein at the neck and at the liposome membrane away from the neck, *I*_GUV_^lipid^ and *I*_neck_^lipid^ represent the fluorescence intensities of the protein at the neck and at the liposome membrane away from the neck, and *I*_background_^lipid^ and *I*_background_^protein^ represent the intensities of lipids and protein away from any membrane, respectively.

### DTS Simulation Methodology

To describe large scale membrane conformation, we employed the dynamically triangulated surface simulation technique.^47^ In this approach the molecular details are ignored and a membrane is represented by a dynamically triangulated surface containing *N*_υ_ vertices, *N*_T_ triangles, and *N*_L_ links. DTS being *dynamical* reflects the fact that all the possible triangulations with a given *N*_υ_, *N*_T_, and *N*_L_ can be sampled by sequential flipping of mutual links between neighboring triangles (using a metropolis algorithm). Together with positional updates of the vertices, this gives a fluid character of DTS with full translational invariance in the plane of the surface. Using a set of discretized geometrical operations, each vertex is furthermore assigned with a unit normal **N̂**_υ_, surface area *A*_υ_, principal curvatures (*c*_1υ_, *c*_2υ_), and principal directions (**X**_1_(υ), **X**_2_(υ)).^48^ The bending elastic energy of the membrane (*E*_b_) is described by Helfrich Hamiltonian that is expressed in the terms of the mean curvature, *H* = 0.5(*c*_1_ + *c*_2_), and Gaussian curvature, *K* = *c*_1_*c*_2_. A discretized form of the Helfrich Hamiltonian is written as
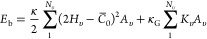
1where κ is the bending modulus, κ_G_ is the Gaussian modulus, *C̅*_0_ is the spontaneous curvature which represents a possible asymmetry between the two monolayers (*C̅*_0_ = 0 for a symmetric membrane). The second term of this equation only depends on the surface topology and does not change by continuous membrane deformation (Gauss-Bonnet theorem).

To model the effect of osmotic pressure we start with the Jacobus van’t Hoff equation Π = *icRT*, where *i* is the van’t Hoff index, *c* is the molar concentration of solute, *R* is the ideal gas constant, and *T* is the temperature. For a vesicle system, we define, *V*_ini_ as the initial volume of the vesicle and effective compartment concentration of solute as , where *j* is either inside (at *V*_ini_) or outside and the summation runs over all the solute types in the *j* compartment. Therefore, we have
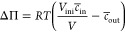
2The energy associated with changes of the vesicle volume from *V*_ini_ to *V* is obtained as
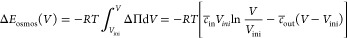
3
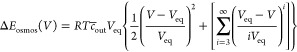
4where *c̅*_out_*V_eq_* = *c̅*_in_*V*_ini_ and *K* = *RTc̅*_out_*V*_eq_. In our experiments, *c̅*_out_ and *c̅*_in_ are in the order of 10s of mM, and the vesicle volume is *V* ∼ μm^3^, therefore, *K* ∼ 10^5^–10^6^*kT* that is much larger than bending energy associated with vesicle deformations at this scale (4πκ ∼ 10^2^–10^3^*kT*). Therefore, only the first term of eq 4 will be relevant and the vesicles will adopt to 

In our simulations, we use the first term of eq 4 for controlling the volume
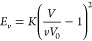
5where  (i.e., the volume of a perfect sphere with the area of *A*) and *v* is the targeted scaled volume. Note that *v* ≤ 1, and *v* = 1 gives a spherical vesicle. To compare to the experimental setup, .

We assume that the collective effect of the SMS on the membrane is to impose a fixed total membrane curvature, e.g., the effects of the chol-oligo/nanostars adsorbing on one side of the membrane. We ensure this by coupling the system energy to a potential as
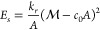
6where , *m*_0_ is average membrane global curvature, and *k*_*r*_ is the coupling constant (we used *k*_*r*_ = 3κ). Note, a similar effect can be reproduced by setting *C̅*_0_ = *c*_0_ in eq 1. Therefore, a membrane is defined by six parameters (*N*_υ_, κ, *k*_*r*_, *c*_0_, *K*, *v*). In all simulations κ = 20*kT*, *K* = 1000*kT*, and *k*_*r*_ = 120*kT* unless stated otherwise.

In our approach, a protein is modeled as an inclusion assigned to a vertex in the triangulation. Each vertex can at most occupy one inclusion, which naturally handles the in-plane excluded volume effect between inclusions. Inclusions can move laterally through updates of the triangulation or by jumps between the neighboring vertices via Kawazaki moves. For inclusions with orientation (see below), the in-plane vector (a vector in the plane of its vertex) orientation will be updated through the metropolis algorithm. Since the membrane bending energy in eq 1 only include first and second order of principal curvatures, the proteins-membrane interaction couples to membrane curvature up to second order. FTsZ and Dynamin A are modeled as elongated inclusions that induces local different membrane curvature in different directions. For this, the energy of a vertex containing an inclusion will be elevated by
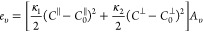
7where *C*^∥^ and *C*^⊥^ are membrane curvature in the direction parallel and perpendicular to the protein orientation that is obtained using the Euler curvature formula; *C*_0_^∥^ and *C*_0_^⊥^ are the preferred membrane curvature of the inclusion in the direction parallel and perpendicular to its orientation, and κ_1_ and κ_2_ are the coupling modules to penalize deviation from the preferred curvatures.^47^ This model has been previously used for FTsZ filaments and banana shape proteins such as BAR protein family.^49,50^ For in-plane symmetric proteins or proteins such as Shiga and cholera toxins,^51^ membrane–protein interaction is modeled as
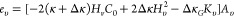
8where *C*_0_, Δκ, and Δκ_G_ are spontaneous curvature of the protein, increase in local membrane bending rigidity due to the protein binding, and increase in local Gaussian membrane rigidity, respectively. Note, only for Δκ_G_ = 0, the membrane curvature imprint of an isolated protein will be equal to *C*_0_. Our results show that such a model does not describe the behavior of FTsZ and Dynamin A observed in our experiments (Figure S16). With the exception of a few cases (Figures S17–S20) when the protein-induced curvature is comparable to vesicle curvature, all the elongated proteins do cluster in the membrane neck. Therefore, we envision FtsZ:ZipA complexes and DynA bind to membranes as elongated proteins, reflecting the nonsymmetric nature of these proteins. Therefore, an elongated protein is defined by four parameters (κ_1_, κ_2_, *C*_0_^∥^, *C*_0_^⊥^) and a symmetric one with three parameters (Δκ, Δκ_*G*_, *C*_0_). In all simulations κ_1_ = 10*kT* and κ_2_ = 0 unless stated otherwise.

For protein–protein interactions, we consider a short-range interaction that is nonzero when a pair of inclusions is residing on the neighboring vertices. This interaction is a function of angle between their in-plane orientations alongside geodesic direction: ΔΘ = Θ_*i*_ – Θ_*j*_′, where Θ_*i*_ is the orientation of inclusion residing on vertex *i*, and Θ_*j*_′ represents the orientation of inclusion residing on vertex *j* after parallel transport to vertex *i.* By keeping the first and second relevant terms, we can write this energy as
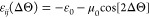
9where −ε_0_ models the isotropic part of the interaction, while the second term models anisotropic interactions.^47^ In the current simulations, when interactions are taken into account, we assume ε_0_ = μ_0_ = *kT*.

### DTS Simulation Setup

DTS simulations were performed on two triangulated meshes that differ in their sizes: a big and a small one, each containing 2452 and 802 vertexes, respectively. We used the smaller system to reach higher sampling. To be consistent with experimental systems, we report a positive/negative *C*_0_, *C*_0_^∥^, or *C*_0_^⊥^ as bending toward/inward the side where the protein is bound. However, our model does not distinguish the side of the membrane. For instance, a protein with positive *C*_0_^∥^, bound from the inside, has the same effect as a negative *C*_0_^∥^ bound from the outside. The model parameters are given in Table S6. For each parameter set at least 5 different replicas were considered. All simulations were performed for 5 × 10^6^ Monte Carlo sweeps. A sweep corresponds to *N*_ν_ attempts to move vertices and *N*_L_ attempts to flip links, *N*_p_ attempts to perform Kawasaki or in-plane rotation of the proteins (details of these moves can be found in refs (33) and (50)).

### MD Simulations

To investigate the interaction of the chol-oligo (cholesterol+ssDNA) with the membrane, a Martini coarse-grained model was made.^21^ The ssDNA anchor strand was built atomistically (http://www.scfbio-iitd.res.in/software/drugdesign/bdna.jsp) and coarse-grained using martinize-dna.^52^ The soft single strand DNA settings were used for the construction of the elastic network. An additional bond was introduced between the first beads of the cholesterol (ROH) and the ssDNA (BB2) using a bond length of 0.470 nm and a force constant of 1250.0 kJ/(mol nm^2^). A small POPC (palmitoyl-oleoyl-*sn*-glycero-3-phosphocholine) bilayer patch with the anchor was generated using the *insane* tool.^53^ POPC, rather than the DOPC used in the experimental setup, was chosen to cleanly study the curvature-inducing effect of chol-oligo, as POPC only has a small intrinsic curvature. To explicitly probe the wedging role of the ssDNA strand rather than the obvious wedging effect of asymmetrically inserting cholesterol, additional cholesterol molecules^53,54^ were added to obtain a balanced system. The upper leaflet contained 1 anchor, 15 POPC lipids, and 2 cholesterols. The lower leaflet contained 15 POPCs and 3 cholesterols. In addition to the membrane components the small box contained 1566 CG waters, 175 antifreeze particles, 34 sodium ions, and 20 chloride ions (0.157 mM NaCl and neutralizing ions). The standard mdp settings for Martini with Verlet were used.^55^ The box was energy minimized for 10,000 steps using the steepest descent algorithm and the GROMACS molecular dynamics toolkit. Equilibration was performed for 50,000 steps with a time step of 5 fs. The v-rescale thermostat^56^ was used, coupling the anchor, cholesterol, POPC, and solvent separately at 310 K (tau_t = 1 ps). The Berendsen barostat was used for semi-isotropic pressure coupling at 1 bar (tau_t = 6 ps) and a compressibility of 3 × 10^–4^ bar^–1^ in both dimensions. For production, we used an increased time step of 10 fs, and the Parrinello–Rahman barostat was used (tau_t = 12 ps). The small bilayer patch was run until stable box dimensions were obtained (<100 ns). To assess the clustering of the chol-oligos, the final configuration was copied 10 times in both lateral dimensions. The resulting large box measured roughly 40 × 40 × 16 nm^3^ and was simulated for 2.4 μs. The cluster analysis was performed using gmx cluster, clustering all ssDNA molecules within 1 nm of each other, over the last 1 μs of the production run. To assess the curvature preference of the chol-oligos, five additional systems were constructed: (i) a flat bilayer consisting of 327 POPC and 13 chol-oligo in each leaflet, where the system was coupled to semiisotropic pressure algorithm; (ii–iv) three systems consisting of 327 POPC and 6, 10, and 13 chol-oligo molecules, respectively, in each leaflet; (v) a control system consisting of 327 POPC and 13 cholesterol molecules. Systems ii–v were compressed in one of the lateral directions to create a buckled membrane and subsequently run at constant area for 1–3 μs with the pressure coupling applied to the perpendicular (*Z*) direction only. Visualization of the simulations was performed using VMD.

## Supporting Information Available

DTS simulations, radial distribution functions in model membranes, simulated curvature indexes, additional characterization of stomatocytes, diffusion models, comparison of different encapsulation methods, simulation of protein clustering aound necks, comparison of SMS with the tube pulling approach, sequences of DNA oligos and compositions of lipid mixes and buffers, captions describing Movies 1–9, and additional references (PDF)
Movie 1: DTS simulation of shape transformation from a sphere to stomatocyte (AVI)
Movie 2: DTS simulation of shape transformation from a sphere to dumbbell (AVI)
Movie 3: Movie of a confocal plane across a stomatocyte that was obtained by the SMS. The arrowhead indicates the position of the neck connecting the inner and outer vesicle in the stomatocyte. Lipid fluorescence is shown in yellow. Frame rate was 1 image/s (AVI)
Movie 4: Movie of a confocal plane across a dumbbell that was obtained by the SMS approach but without the addition of chol-oligo/nanostars. The elliptical and fluctuating shape of the lobes indicates low membrane tension. Frame rate was 1 image/s (AVI)
Movie 5: Movie of a confocal plane across a dumbbell that was obtained by the SMS approach with the addition of chol-oligo/nanostars. The constant spherical shape of the lobes indicates high membrane tension. Frame rate was 1 image/s (AVI)
Movie 6: Movie of a confocal plane across a dumbbell that was obtained by the SMS. One lobe is being bleached and lipid fluorescence recovery is shown. Frame rate was 1 image/s (AVI)
Movie 7: Movie of a confocal plane across a stomatocyte that was obtained by the SMS in the presence of Dynamin A, which forms a cluster at the neck of the stomatocyte. Lipid fluorescence is shown in magenta, Dynamin A is in green. Frame rate was 1 image/s (AVI)
Movie 8: Movie of a confocal plane across a stomatocyte that was obtained by the SMS in the presence of FtsZ + ZipA, which led to formation of an array of inward elongated tubes. Lipid fluorescence is shown in magenta, proteins are in cyan. Frame rate was 1 image/s (AVI)
Movie 9: Movie of a confocal plane across a dumbbell that was obtained by the SMS in the presence of FtsZ + ZipA, which led to formation of an elongated neck connecting the two lobes. Lipid fluorescence is shown in magenta, proteins are in cyan. Frame rate was 1 image/s (AVI)

## Supplementary Material

nn2c06125_si_001.pdf

nn2c06125_si_002.avi

nn2c06125_si_003.avi

nn2c06125_si_004.avi

nn2c06125_si_005.avi

nn2c06125_si_006.avi

nn2c06125_si_007.avi

nn2c06125_si_008.avi

nn2c06125_si_009.avi

nn2c06125_si_010.avi
