# Supplementary material for: Synthetic Membrane Shaper for Controlled Liposome Deformation
Source: ACS Nano. 2022 Nov 28;17(2):966–78. doi: 10.1021/acsnano.2c06125 (PMC9878720; doi:10.1021/acsnano.2c06125)
Supplement: Supplementary file 1 — nn2c06125_si_001.pdf [file nn2c06125_si_001.pdf]

Supplementary material

A synthetic membrane shaper for controlled liposome deformation

Nicola De Franceschi[1], Weria Pezeshkian[2,3], Alessio Fragasso[1], Bart M.H. Bruininks[2], Sean Tsai, Siewert J. Marrink[2], and Cees Dekker[1]

[1] *Department of Bionanoscience, Kavli Institute of Nanoscience Delft, Delft University of Technology, Delft, The Netherlands*

[2] *Groningen Biomolecular Sciences and Biotechnology Institute and Zernike Institute for Advanced Materials, University of Groningen, Nijenborgh 7, 9747 AG Groningen, The Netherlands.*

[3] *The Niels Bohr International Academy, The Niels Bohr Institute, University of Copenhagen, Copenhagen, Denmark*

Table of contents

Supplementary Figure 1: Description and examples of dumbbells and stomatocytes………... 3
Supplementary Figure 2: Vesicle simulations for different reduced volume and spontaneous curvature……………………………………………..……4
Supplementary Figure 3: Radial distribution function……...……………………………..5
Supplementary Note 1: Positive and negative curvature index……………………………….. 6
Supplementary Figure 4: chol-oligo induces wedging……………………………………….. 8
Supplementary Figure 5: PCI, NCI and TC for 26 oligo-CHOL molecules as a function of time………………………………………………………………… 9
Supplementary Figure 6: PCI, NCI and TC for 20 oligo-CHOL molecules as a function of time …………………………………………………………...... 10
Supplementary Figure 7: PCI, NCI and TC for 20 CHOL molecules as a function of time .. 11
Supplementary Figure 8: Quantification of chol-oligo clustering in MD simulation………....12
Supplementary Figure 9: Characterization of stomatocytes………………………………...... 13
Supplementary Note 2: Diffusion model in stomatocyte system……………………………….. 14
Supplementary Figure 10: Example of fitting to diffusion model in stomatocyte system…… 16
Supplementary Figure 11: Example of fitting to diffusion model in stomatocyte produced without nanostars and chol-oligo…………………………………………………………………17
Supplementary Figure 12: Example of stomatocytes obtained without chol-oligo/nanostars...18
Supplementary Figure 13: Chol-oligo/nanostars generate membrane curvature in Dumbbells……………………………………………………………… 19
Supplementary Note 3: Diffusion model in dumbbell system………………………………….. 20

Supplementary Figure 14: Example of fitting to diffusion model in dumbbell system……... 22
Supplementary Figure 15: Comparison of different encapsulation methods for DynA 23
Supplementary Figure 16: Symmetric proteins failed to cluster around the membrane neck ………………………………………………………….... 24
Supplementary Figure 17: Behavior of elongated proteins with different spontaneous curvature around membrane neck ……………………………..... 25
Supplementary Figure 18: Behavior of proteins with different association strength around membrane neck.…………………………………………….......27
Supplementary Figure 19: Stomatocyte system robustness upon changing protein parameters………………………………………………………….. 28
Supplementary Figure 20: Dumbbell system robustness upon changing protein parameters .29
Supplementary Table 1: Average value for PCI, NCI and TC……………………….............30
Supplementary Table 2: Comparison between the tube pulling and SMS approaches.............31
Supplementary Table 3: DNA sequences used……………………………………...............32
Supplementary Table 4: Inner solutions…………………………………………..................33
Supplementary Table 5: Outer solutions………………………………………................... 34
Supplementary Table 6: Lipid mixes composition (expressed in %mol)………..….................. 35
Movie captions……………………………………………………………………...............36
References…...……………………………………………………………………...............37

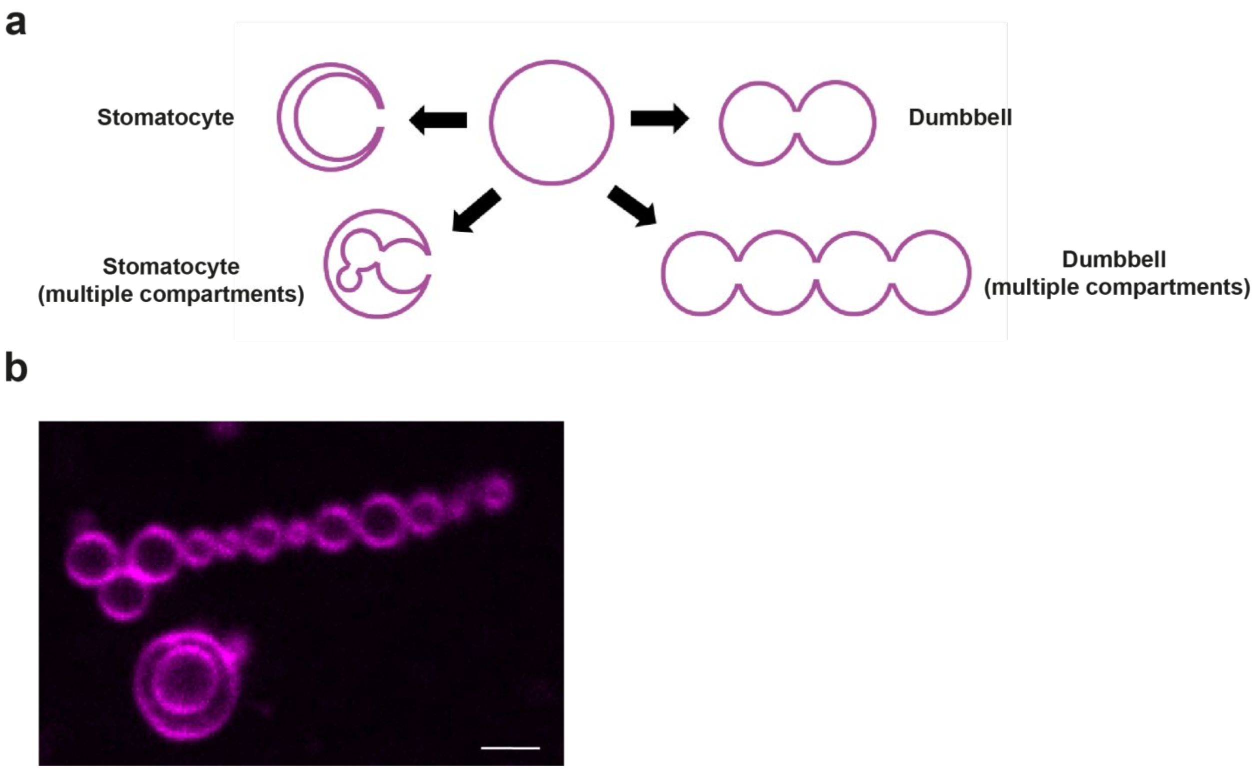

Supplementary Figure 1: Description and examples of dumbbells and stomatocytes (a) Schematics depicting membrane shape transformations of a spherical liposome into dumbbells and stomatocytes. In both cases, multiple compartments can originate from the same liposome. **(b)** Example of coexistence of dumbbells and stomatocytes in a liposome preparation obtained by the SMS approach by omitting nanostars and chol-oligos. Scale bar: 5µm.

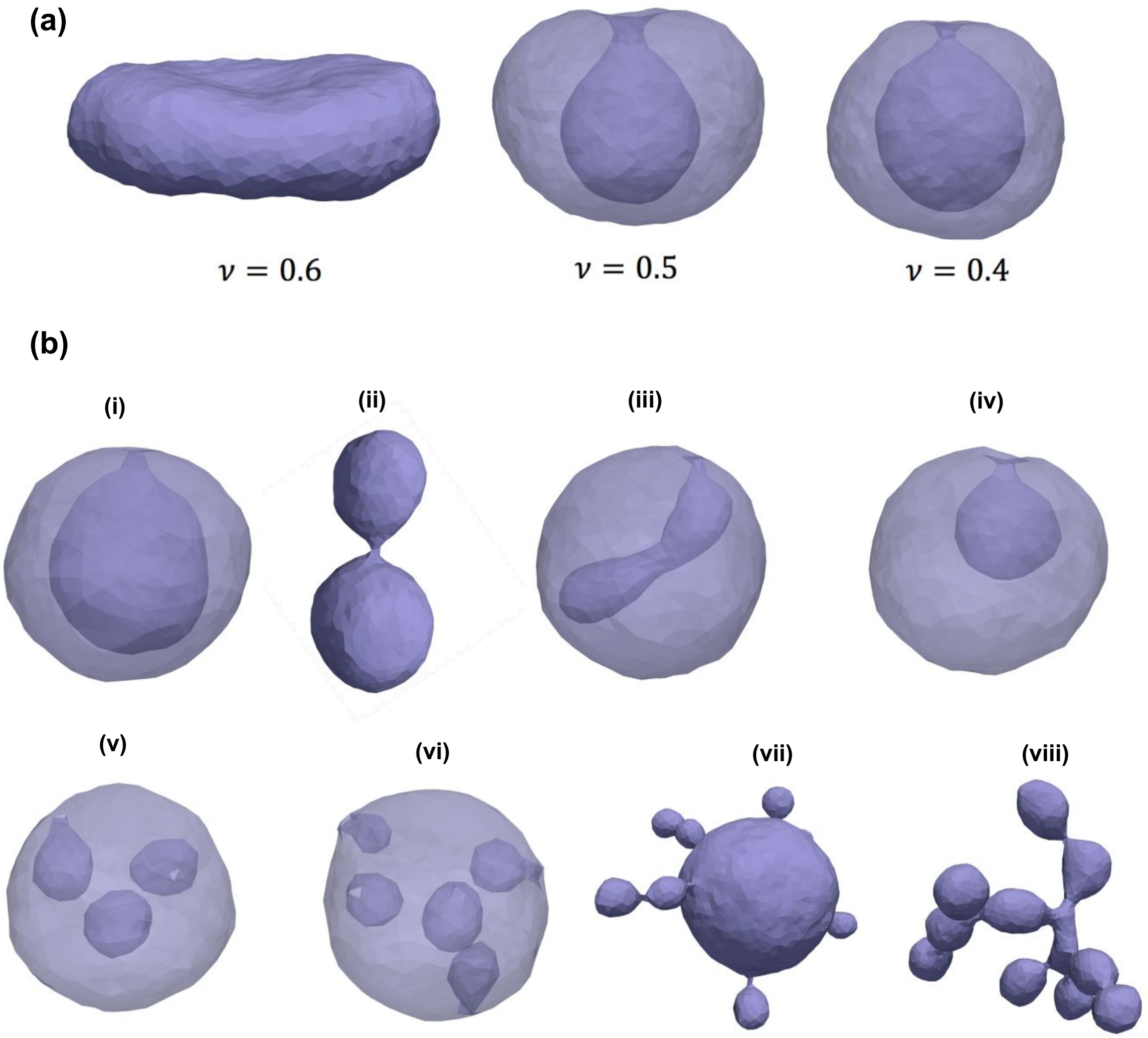

Supplementary Figure 2: DTS simulations for different reduced volume and spontaneous curvature. **(a)** Oblate- to-stomatocyte transition as a function of reduced volume ν, only a limited number of different shapes can be obtained by reducing the vesicle volume. **(b)** Diverse ranges of vesicle shape obtained from membrane spontaneous curvature (c_0). c_0 is reported in the unit of a^{-1} and R^{-1}; where a is the characteristic length of the DTS model and R=$\sqrt{A/4\pi}$; the radius of the vesicle with area of A if it was spherical. Number of vertices (N_υ) for i-vi is 1060 and for $N_\upsilon = 2452$ for vii-viii (i) No volume coupling, $c_0 = -0.1[a^{-1}] = -1.2[R^{-1}]$ (ii) $\upsilon = 0.7, c_0 = 0.3[a^{-1}] = 3.6[R^{-1}]$ (iii) $\upsilon = 0.7, c_0 = -0.1[a^{-1}] = -1.2$ $[R^{-1}]$ (iv) $\upsilon = 0.7, c_0 = -0.03[a^{-1}] = -0.36[R^{-1}]$ (v) $\upsilon = 0.6, c_0 = -0.1[a^{-1}] = -1.2[R^{-1}]$ (vi) $\upsilon = 0.6, c_0 = -0.2[a^{-1}] = -2.4[R^{-1}]$ (vii) $\upsilon = 0.6, c_0 = 0.5\ [a^{-1}] = 7.5[R^{-1}]$, (viii) No volume coupling, $c_0 = 0.5\ [a^{-1}] = -7.5[R^{-1}]$.

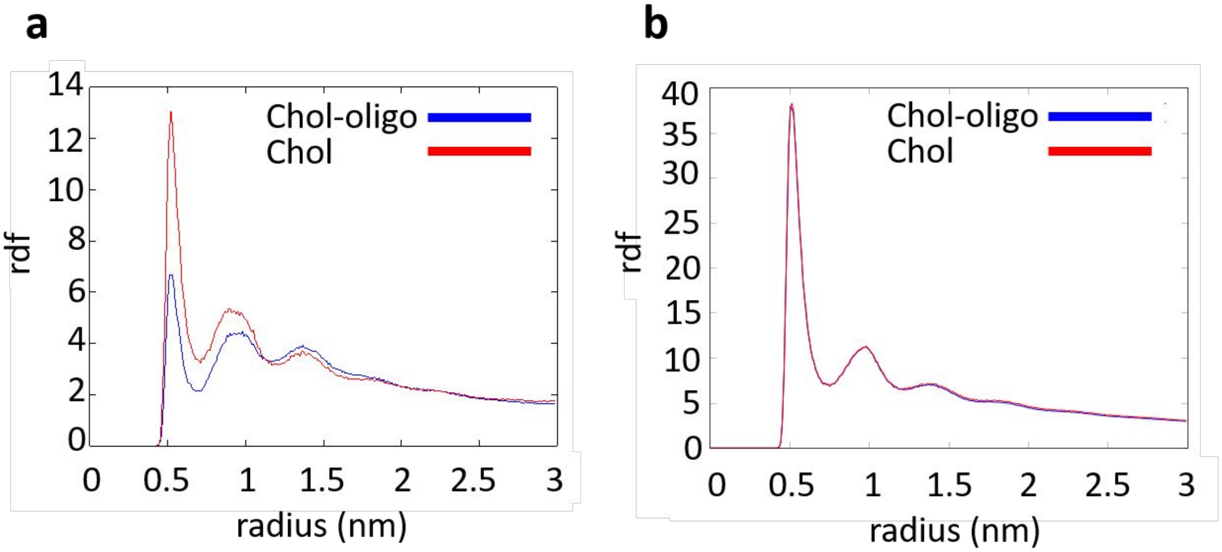

Supplementary Figure 3: Radial distribution function (rdf) from MD simulations. The membranes analyzed are composed of either POPC/cholesterol ('Chol', red), or POPC/chol-oligo ('Chol-oligo', blue). **(a)** Radial distribution function for the POPC head group (phosphate) taken with respect to the polar group (hydroxyl) of the cholesterol moiety. The decrease of the first and second peaks in the system containing chol-oligos, compared to the system containing cholesterol only, shows that the chol-oligos give rise to reduced packing at the lipid headgroup level, i.e., causing expansion. This is also reflected in the increase in the total bilayer area (Supplementary Note 1). **(b)** Radial distribution function of POPC terminal beads taken with respect to the terminal bead of the cholesterol moiety. The strong overlap of the rdfs shows that the chains are equally densely packed for both systems. Together, the decrease in the first peak of the rdfs in the interfacial region in panel (a) and the lack of an effect deeper in the membrane (b) point to a wedging effect of chol-oligo (see Figure S4).

Supplementary Note 1 - Positive and negative curvature index

A bilayer containing 13 oligo-chol in both leaflets showed an increase of 3.7 $\pm$ $0.1 nm^2$ in the projected area compared to the one containing 13 chol molecules. We thus estimate the increase in projected area per molecule of a chol-oligo versus chol to be $\delta a = 0.28\, nm^2$. Radial distribution functions of the POPC head group with respect to the head group of chol moiety showed a significant decrease of the first peak for the oligo-chol system (Figure S3a), whereas such a decrease was not observed for the terminal beads (Figure S3b) – which indicated that the δa came from an expansion in the head region.

Based on these results we can make a rough estimate of the curvature ΔC induced by a single oligo-chol compared to the one induced by chol, see Figure S4. In this system, $\Delta A = A_{up} - A_{mid} \sim 2hA_{mid}/R$ for $R \gg h$ (or small curvature), since $A_{up} = \pi(R+h)^2 \sin^2(\frac{\theta}{2})$ and $A_{mid} = \pi R^2 \sin^2(\frac{\theta}{2})$. Therefore, the curvature of the midplane will be

$$C = \frac{2}{R} = \frac{\Delta A}{hA_{mid}} \quad . \quad (1)$$

As we do not know the exact value of ΔA, and therefore, we obtain C with respect to a reference point which is the curvature of the chol molecule. The RDF of the tail bead suggest that A_{mid} for cholesterol is equal to A_{mid} for chol-oligo. Therefore

$$\Delta C = C_{oligo} - C_{chol} = \frac{\delta a}{h[APL_{chol}]} \sim 0.3\, nm^{-1} \,. \qquad (2)$$

To systemically investigate if chol-oligo can lead to curvature generation, we introduce three membrane curvature identifiers; positive curvature index (PCI), negative curvature index (NCI), and total curvature (TC: in nm^{-1}), which is the sum of the two. The PCI measures an average of the number of instances that a molecule resides on the part of the membrane with positive curvature. PCI=1 indicates that the molecule has no preference for positive or negative curvature while a value larger than one indicates that the molecule prefers positive curvature. NCI is the same measure but for negative curvature. TC is the average value of the membrane curvature where the molecule is located.

$$\mathrm{PIC} = \frac{N_{pos}A}{NA_{pos}}$$

$$\mathrm{NIC} = \frac{N_{neg}A}{NA_{neg}}$$

$$A_{neg} + A_{pos} = A$$

$$N_{neg} + N_{pos} = N$$

Where N_{pos} ($N_{neg/pos}$) is the number of molecules located on membrane regions with positive/negative mean curvature, $A_{pos}(A_{neg/pos})$ is the area of the membrane that has positive/negative mean curvature, A is the total membrane area, and N is the total number of the molecules. Figure S5 shows the time evolution of these quantities. It is clear that chol-oligo is associated with positive curvature.

To further explore this association, we performed simulations of POPC membranes containing different compositions of chol-oligo. In these simulations, we fixed the lateral (XY) box size to 20 nm × 10 nm. The results show that a system containing 20 chol-oligo has a PCI of 1.5 while for the 20 cholesterol system this value is 0.79. These results indicate that cholesterol prefers negative curvature while chol-oligo prefers positive curvature (Figure S6, S7). Interestingly, PCI is directly correlated to the chol-oligo concentrations (Table S1).

Overall, we envision two important roles for the oligo moiety of the construct for the generation of membrane curvature; i) making a large head group for an amphiphilic chol-oligo construct, leading to a wedging effect, ii) being a highly charged and polar group and preventing the construct from flipflopping. These lead the chol-oligo bound monolayer to prefer a bent shape.

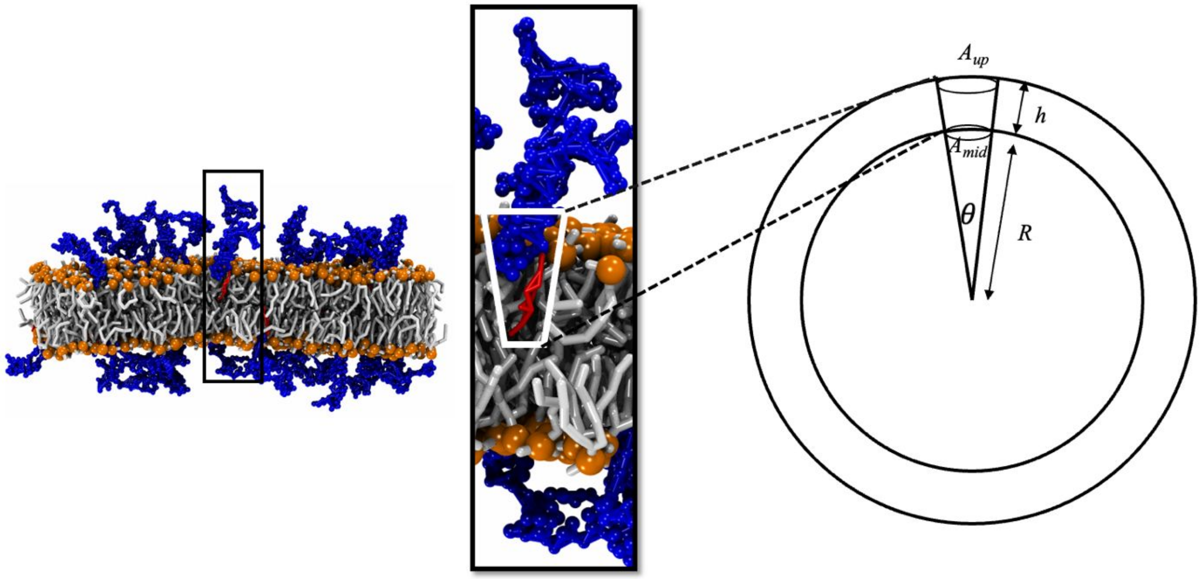

Figure S4: chol-oligo induces wedging. The effect of a single chol-oligo molecule can be seen as the intersecting volume of a spherical shell with a cone shown in the right side.

,

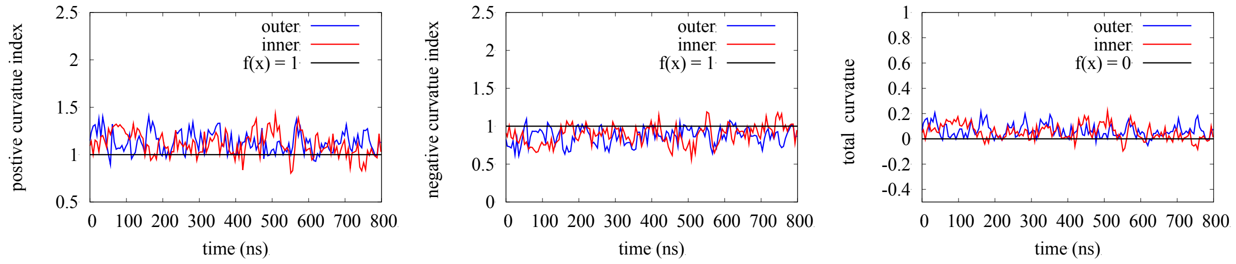

Supplementary Figure 5: PCI, NCI and TC as a function of time for a flat bilayer consisting of 327 POPC and 13 chol-oligo in each leaflet . For the average value, see Table 1 “13 chol-oligo/flat” row. All three measures of curvature indicate that chol-oligo are associated with positive mean curvature.

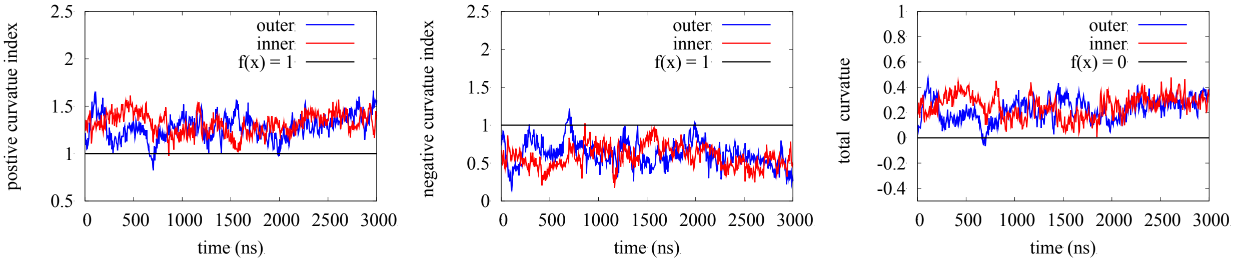

Supplementary Figure 6: PCI, NCI and TC as a function of time for a buckled bilayer consisting of 327 POPC and 10 chol-oligo in each leaflet. For the average value, see Table 1 "10 chol-oligo/flat" row. Buckling amplifies the molecules curvature behaviors as the membrane can exhibits large curvature. All three measures of curvature indicate that chol-oligo are associated with positive mean curvature.

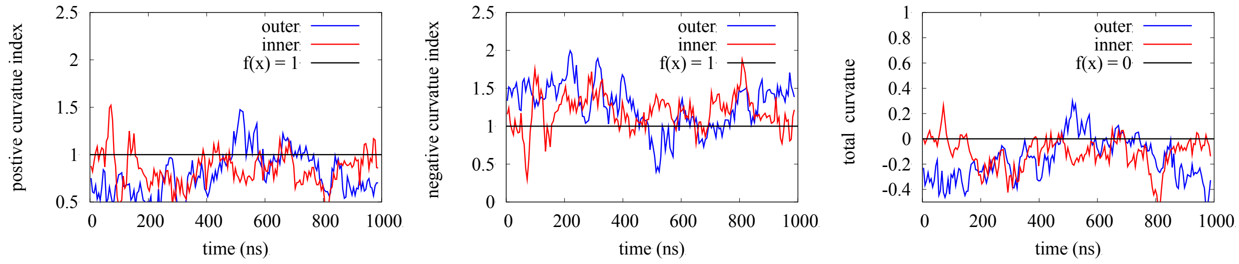

Supplementary Figure 7: PCI, NCI and TC as a function of time for a buckled bilayer consisting of 327 POPC and 10 cholesterol in each leaflet (a control system). For the average value, see Table 1 “10 chol” row. All three measures of curvature indicate that chol induces negative curvature and therefore the positive curvature of chol-oligo does not come from the chol moiety of the construct (see Figure SI6).

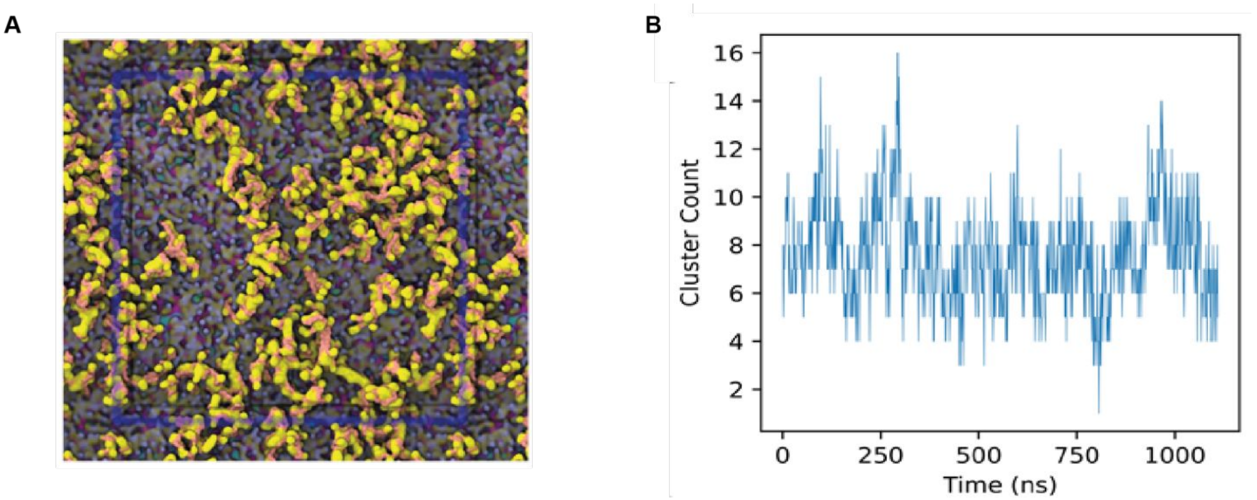

Supplementary Figure 8: Quantification of chol-oligo clustering in MD simulation. (a) Top view snapshot of an MD simulation of multiple chol-oligos inserted into the POPC/cholesterol membrane. **(b)** Clustering analysis of chol-oligos inserted into the membrane, performed by counting the number of chol-oligos present within 1 nm of each other over the last 1 μs of the simulation. The constant change in the count displayed indicates that no significant stable clustering is occurring.

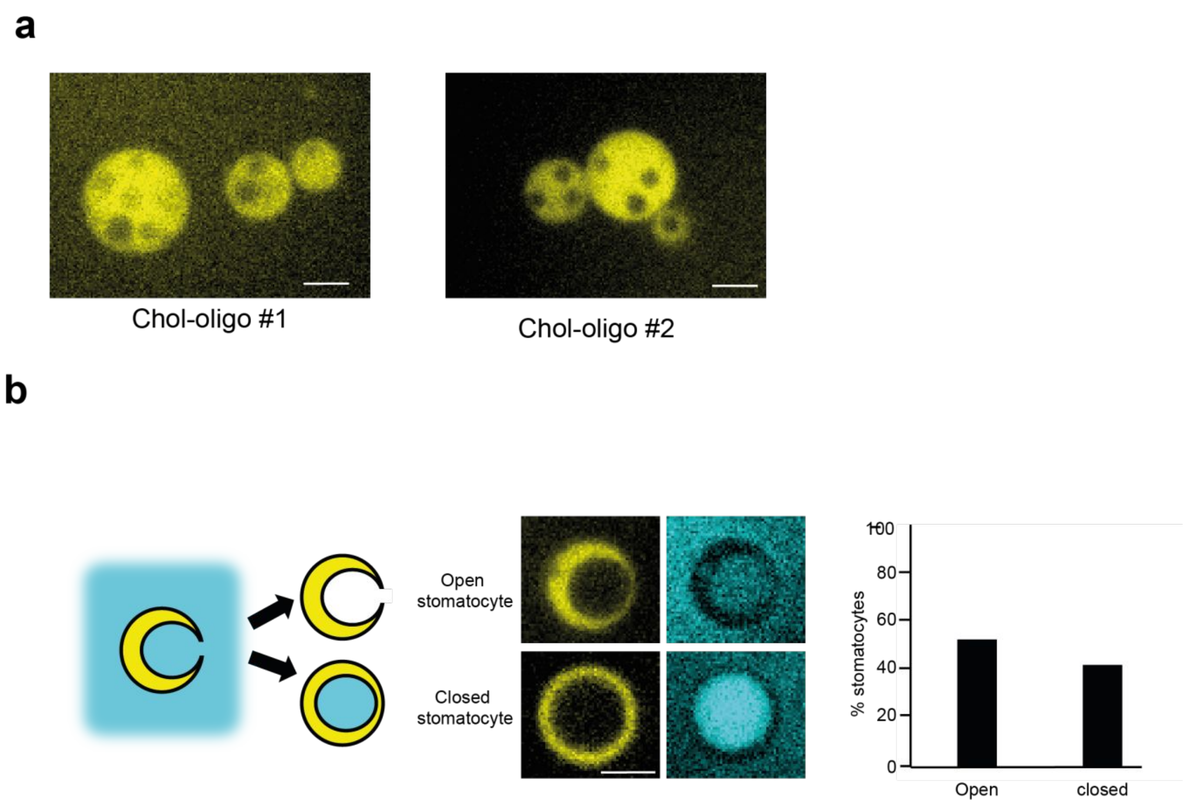

Supplementary Figure 9: Characterization of stomatocytes. (a) Morphology of stomatocytes that were produced using chol-oligomers of different sequences. No significant sequence dependence was observed. Chol-oligo#1 is 15 bases for oligo#1 chol-oligo#2 is 35 bases long. **(b)** Quantification of the fraction of open versus closed stomatocytes in a SMS preparation. Stomatocytes were produced in the presence of the small soluble fluorescent dye Aleaxa-647 (in cyan), which was encapsulated in the internal compartment during the formation of stomatocytes. The sample was then diluted so that the dye could diffuse out of the lumen via the pore. Therefore, only closed stomatocytes could retain the dye. The intermembrane space is visualized by encapsulation of Alexa-555 (in yellow). N=171 stomatocytes from 2 independent preparations were quantified. Scale bars: 5µm.

Supplementary Note 2 – Diffusion model in a stomatocyte geometry

We model bulk diffusion across the neck of a stomatocyte as follows:

a) A vesicle is connected by a channel of length L_p to the outer aqueous solution (OA).
b) The inner aqueous (IA) solution of the vesicle starts with a much lower concentration of fluorescent molecules, due to photobleaching step, compared to OA, namely $\varphi_{IA,start} \ll \varphi_{OA,start}$, where $\varphi_{IA,start}$ and $\varphi_{OA,start}$ are the initial concentrations of fluorescent molecules present in IA and OA, respectively.
c) $\varphi_{OA}(t) = \varphi_{OA}$, i.e., the concentration of molecules in the OA is constant over time.

The derivation follows the same steps as described in Ref.1. Let us first define the flux ϕ which represents the net flux of molecules going from OA to IA. According to the first Fick's law:

$$\phi = -D\frac{d\varphi}{dx} \quad , \quad (1)$$

where $\frac{d\varphi}{dx}$ is the concentration gradient, and D is the diffusion constant of the molecule. By definition of flux, namely the number of molecules dN per unit of surface dS per unit of time dt, we have that

$$\phi = \frac{dN}{dSdt} \quad . \quad (2)$$

Combining (1) and (2) we obtain

$$\frac{dN}{dSdt} = -D\frac{d\varphi}{dx} \quad , \quad (3)$$

and integrating over a finite area A_p (i.e., the cross-sectional area of the pore), and length L_p (length of the pore) we find

$$\frac{dN}{dt} = A_p D\frac{\varphi_{OA} - \varphi_{IA}(t)}{L_p} \quad , (4)$$

where $\frac{dN}{dt}$ is the influx rate, which is the number of molecules with diffusion constant D crossing a pore of length L_p and area $A_p = \frac{\pi}{4}{d_p}^2$ ($d_p =$ pore diameter) per unit of time dt. We can write the molecular concentration in the IA volume as $\varphi_{IA}(t) = \frac{N_{IA}(t)}{V}$, where V is the volume of vesicle. We also note that the total number of molecules in OA is constant over time, as the volume of the outer solution is much larger (~200 uL) than the ~picoliter volume of the vesicle. We can rewrite for as a function of $\varphi_{IA}(t)$,

$$\frac{d\varphi_{IA}(t)}{dt} = \frac{A_p D}{V}\frac{\varphi_{OA}(t) - \varphi_{IA}(t)}{L_p} , \quad (5)$$

which, with $\varphi_{IA}(t = 0) = \varphi_{IA,start}$, has a solution

$$\varphi_{IA}(t) = \varphi_{OA} - (\varphi_{OA} - \varphi_{IA,start})\, e^{-\frac{A_p D}{V L_p}t} \quad . \quad (6)$$

Upon rearranging Eq.6, we then obtain

$$\frac{\varphi_{OA} - \varphi_{IA}(t)}{\varphi_{OA}} = (1 - \frac{\varphi_{IA,start}}{\varphi_{OA}})\, e^{-\frac{A_p D}{V L_p}t} \quad , \quad (7)$$

where V,$\varphi_{IA}(t)$,φ_{OA} are measured by fluorescence imaging (assuming $\varphi \propto$ intensity), L_p is assumed to be equal to the pore diameter, $D = 330\frac{\mu m^2}{s}$ for Alexa-647 dye as reported by Ref.2, or $D = 435\frac{\mu m^2}{s}$ for Alexa488 dye as reported in Ref.3. This equation is employed to fit the FRAP data with two fit parameters $\frac{\varphi_{IA,start}}{\varphi_{OA}}$ and A_p. This yields the pore area A_p, from which we then estimate the corresponding pore diameter $d_p = 2\sqrt{\frac{A_p}{\pi}}$.

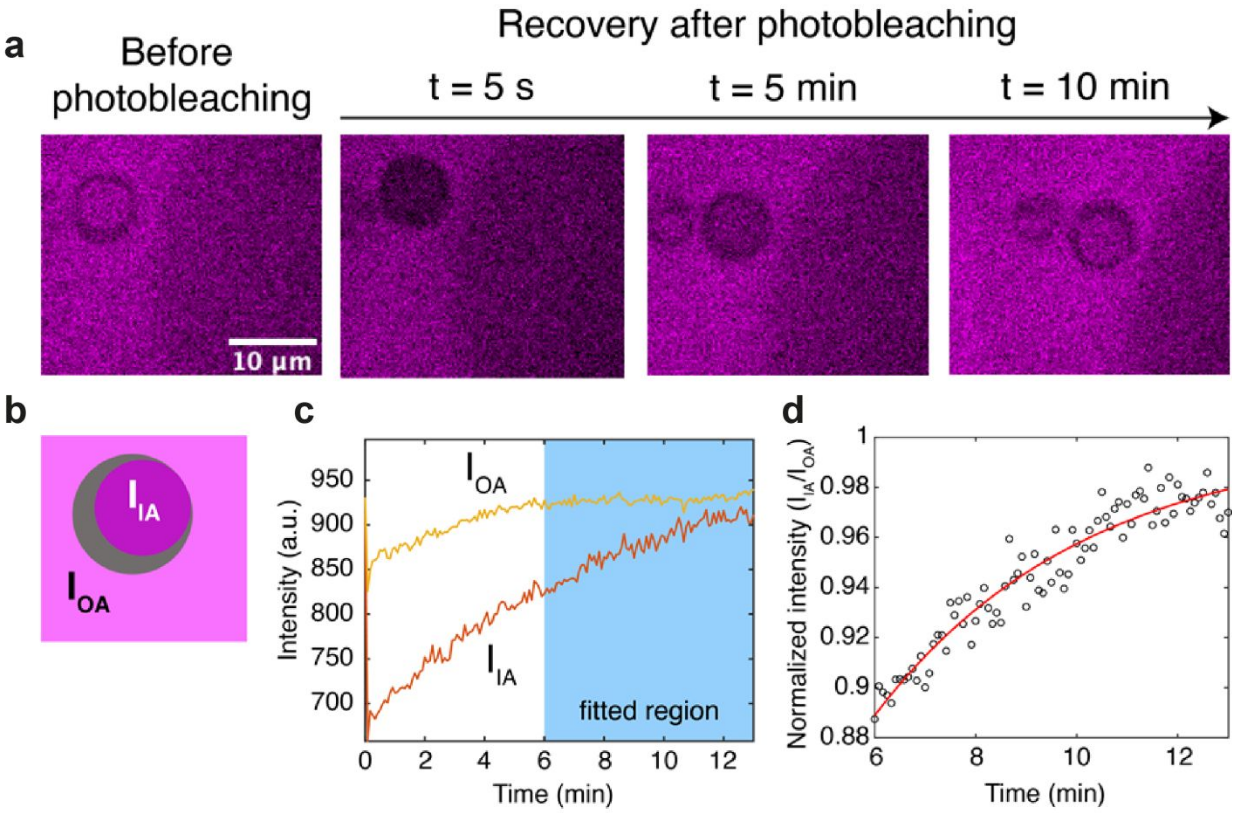

Supplementary Figure 10: Example of fitting to diffusion model in stomatocyte system. (a) FRAP series of images showing a stomatocyte before and after photobleaching of the inner aqueous compartment (IA), followed by recovery. Fluorescence signal was from Alexa647-maleimide. **(b)** Schematic of the stomatocyte system showing the regions of mean fluorescence intensities I_{IA} and I_{OA}. **(c)** Fluorescence intensities I_{IA}, I_{OA} *vs* time. Full recovery, namely when $I_{IA} \approx I_{OA}$, is observed within a timescale of ~10 min. Blue area represents where fitting was performed, as I_{OA} reached an equilibrium value after an initial transient. **(d)** Fitting of Equation (7) to the normalized intensity data $\frac{\varphi_{OA} - \varphi_{IA}(t)}{\varphi_{OA}}$. From this fit, a pore diameter of 24.9 nm was estimated.

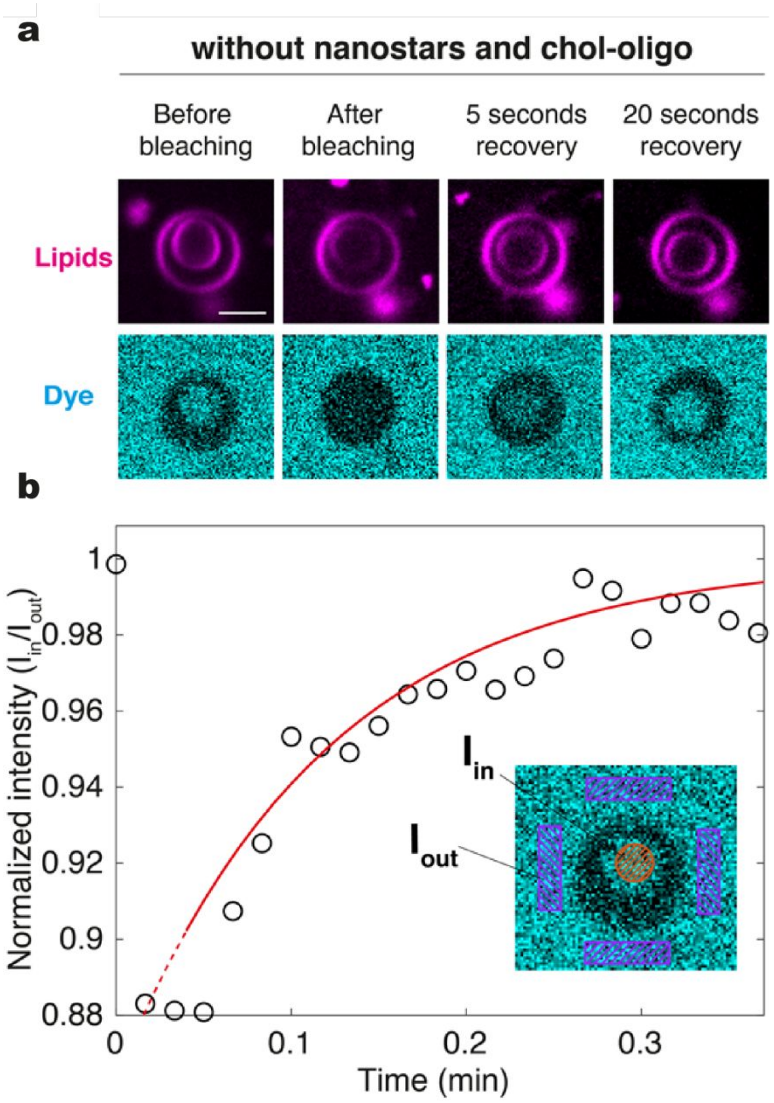

Supplementary Figure 11: Example of fitting to diffusion model in stomatocyte that was produced without nanostars and chol-oligo. (a) FRAP series showing a stomatocyte before and after photobleaching of the inner aqueous compartment (IA), followed by recovery. Fluorescence signal was from Alexa-488-maleimide. **(b)** Solid line denotes fitting of Equation (7) to the normalized intensity data $\frac{\varphi_{OA} - \varphi_{IA}(t)}{\varphi_{OA}}$.

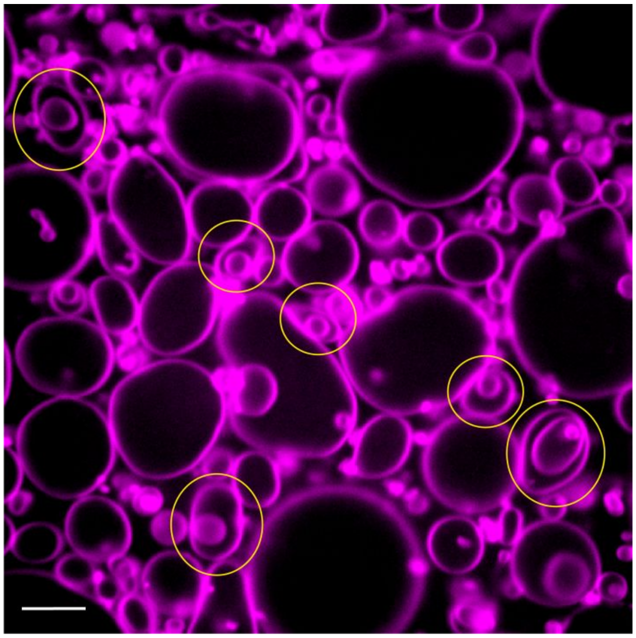

Supplementary Figure 12: Example of stomatocytes obtained without chol-oligo/nanostars. The stomatocytes, which are mostly non-spherical, are indicated by yellow circles. scale bar: 10µm.

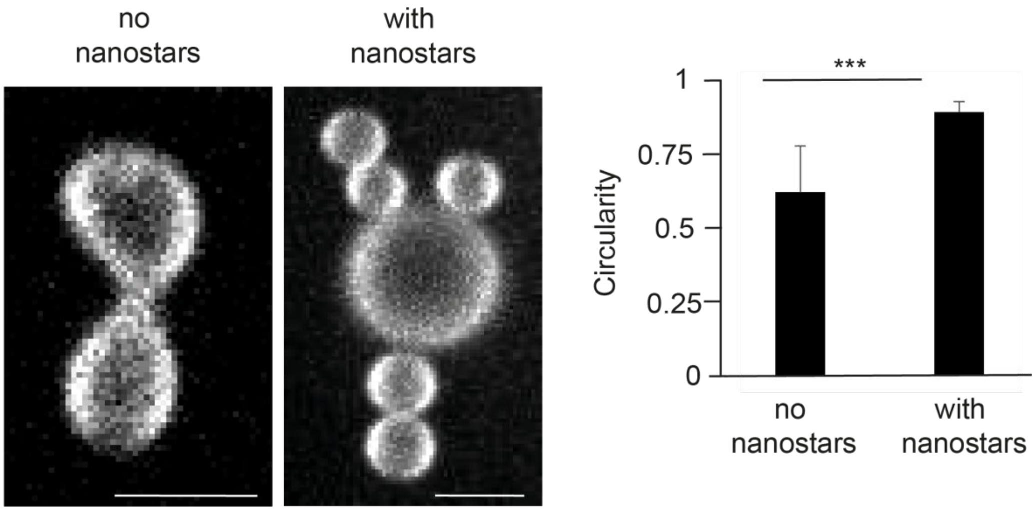

Supplementary Figure 13: chol-oligo/nanostars generate membrane curvature in dumbbells. Quantification of lobe circularity in dumbbells obtained with or without chol-oligo/nanostars. The lobe circularity plotted on the right is calculated from individual frames of time-lapse movies of dumbbells. N=72 frames from 6 dumbbells without chol-oligo/nanostars and n=48 frames from 4 dumbbells with chol-oligo/nanostars where plotted. Scale bar: 5µm.

Supplementary Note 3 – Diffusion model in a dumbbell geometry

We model bulk diffusion across the neck of a dumbbell as follows:

a) Two vesicles are connected by one channel of length L_p and diameter d_p.

b) The first vesicle (V_1) starts with a much lower amount of fluorescent molecules, due to photobleaching step, compared to the second vesicle (V_2), namely $\varphi_{1,start} \ll \varphi_{2,start}$, where $\varphi_{1,start}$ and $\varphi_{2,start}$ are the initial concentrations of fluorescent molecules present in V_1 and V_2, respectively.

c) The total number of fluorescent molecules N_{tot} present in the dumbbell system (after photobleaching) is constant over time.

We define the flux ϕ which represents the net flux of molecules going from V_2 to V_1. According to the first Fick's law:

$$\phi = -D\frac{d\varphi}{dx} \quad , \quad (8)$$

where $\frac{d\varphi}{dx}$ is the concentration gradient, and D is the diffusion constant of the molecule. By definition of flux, namely number of molecules dN per unit of surface dS per unit of time dt, we have that

$$\phi = \frac{dN}{dSdt} \quad . \quad (9)$$

Combining (8) and (9) we obtain

$$\frac{dN}{dSdt} = -D\frac{d\varphi}{dx} \quad , \quad (10)$$

and integrating over a finite area A_p (cross-sectional area of the pore), and length L_p (length of the pore) we find

$$\frac{dN}{dt} = -A_p D\frac{\varphi_2(t) - \varphi_1(t)}{L_p} \quad , \ (11)$$

where $\frac{dN}{dt}$ is the influx rate, i.e., the number of molecules with diffusion constant D crossing a pore of length L_p and area $A_p = \pi {R_p}^2$ ($R_p =$ pore radius) per unit of time dt. We can write the molecule concentration in V_1 as $\varphi_1(t) = \frac{N_1(t)}{V_1}$, where V_1 is the volume of V1. We also note that the total number of molecules N_{tot} in V_1 and V_2 is constant over time. We end up with the following system of equations:

$$\begin{cases} \frac{d\varphi_1(t)}{dt} = -\frac{A_p D}{V_1}\frac{\varphi_2(t) - \varphi_1(t)}{L_p} & (12) \\ N_{tot} = \varphi_1(t)V_1 + \varphi_2(t)V_2 & (13) \end{cases}$$

We can rewrite (13) and express $\varphi_2(t)$ as a function of $\varphi_1(t)$

$$\varphi_2(t) = \frac{N_{tot} - V_1\,\varphi_1(t)}{V_2} \quad .$$

We can substitute the expression for $\varphi_2(t)$ in (12) and rewrite $\frac{d\varphi_1(t)}{dt}$ as a function of $\varphi_1(t)$ only, finding

$$\frac{d\varphi_1(t)}{dt} = -\frac{A_p D}{V_1} \frac{\frac{N_{tot} - V_1 \varphi_1(t)}{V_2} - \varphi_1(t)}{L_p} \quad .$$

Rearranging terms, we find

$$\frac{d\varphi_1(t)}{dt} = -\frac{A_p D}{L_p}\left(\frac{V_1 + V_2}{V_1 V_2}\right)\left(\frac{N_{tot}}{V_1 + V_2} - \varphi_1(t)\right) \ , \qquad (14)$$

which for $\varphi_1(t = 0) = \varphi_{1,start}$ has solution

$$\varphi_1(t) = \frac{N_{tot}}{V_1 + V_2} - \left(\frac{N_{tot}}{V_1 + V_2} - \varphi_{1,start}\right) e^{-\frac{A_p D}{L_p}\left(\frac{V_1 + V_2}{V_1 V_2}\right)t} \quad . \qquad (15)$$

To minimize the number of fit parameters, we express (15) as

$$\frac{\varphi_1(t)(V_1 + V_2)}{\varphi_1(t)V_1 + \varphi_2(t)V_2} = 1 + \left(\frac{\varphi_{1,start}(V_1 + V_2)}{\varphi_1(t)V_1 + \varphi_2(t)V_2} - 1\right) e^{-\frac{A_p D}{L_p}\left(\frac{V_1 + V_2}{V_1 V_2}\right)t} \ , \quad (16)$$

where V_1, V_2, $\varphi_1(t)$, $\varphi_2(t)$ are measured by fluorescence imaging (assuming $\varphi \propto$ intensity), $D = 330\frac{\mu m^2}{s}$ as reported by Ref.2, L_p is assumed to be equal to the pore diameter, and where $\varphi_{1,start}$ and A_p are two fit parameters. From the fitted pore area A_p we can then estimate the corresponding pore diameter $d_p = 2\sqrt{\frac{A_p}{\pi}}$.

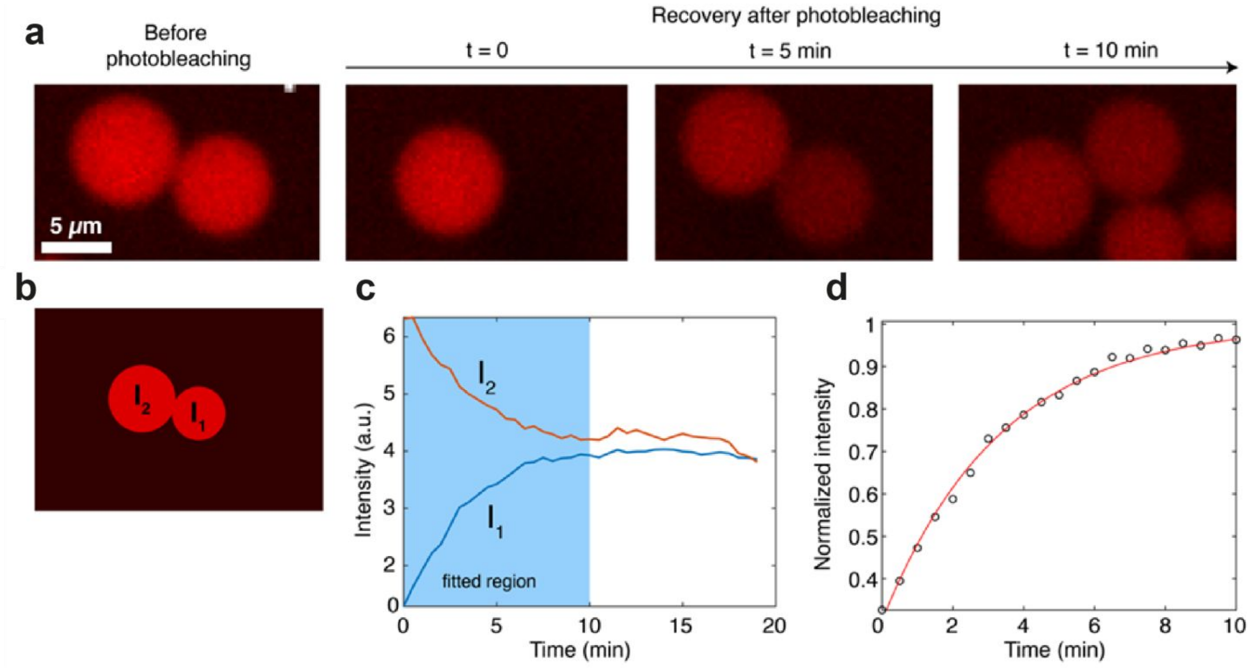

Supplementary Figure 14 – Example of fitting the diffusion model in a dumbbell system (a) FRAP series showing a two-vesicle dumbbell system before and after photobleaching of one of the vesicles, followed by recovery. Fluorescence signal comes from a soluble Alexa647-maleimide dye. **(b)** Schematics of the dumbbell system showing the regions where the measures of mean fluorescence intensities I_1, I_2 were acquired. **(c)** Fluorescence intensities I_1, I_2 *vs* time. Full recovery (namely when $I_1 \approx I_2$) is observed within a timescale of ~10 min. Blue area represents where the fitting was performed. **(d)** Fitting of Equation (16) to the normalized intensity data $\frac{\varphi_1(t)(V_1 + V_2)}{\varphi_1(t)V_1 + \varphi_2(t)V_2}$. From the fit, a pore diameter of 8.6 nm was estimated.

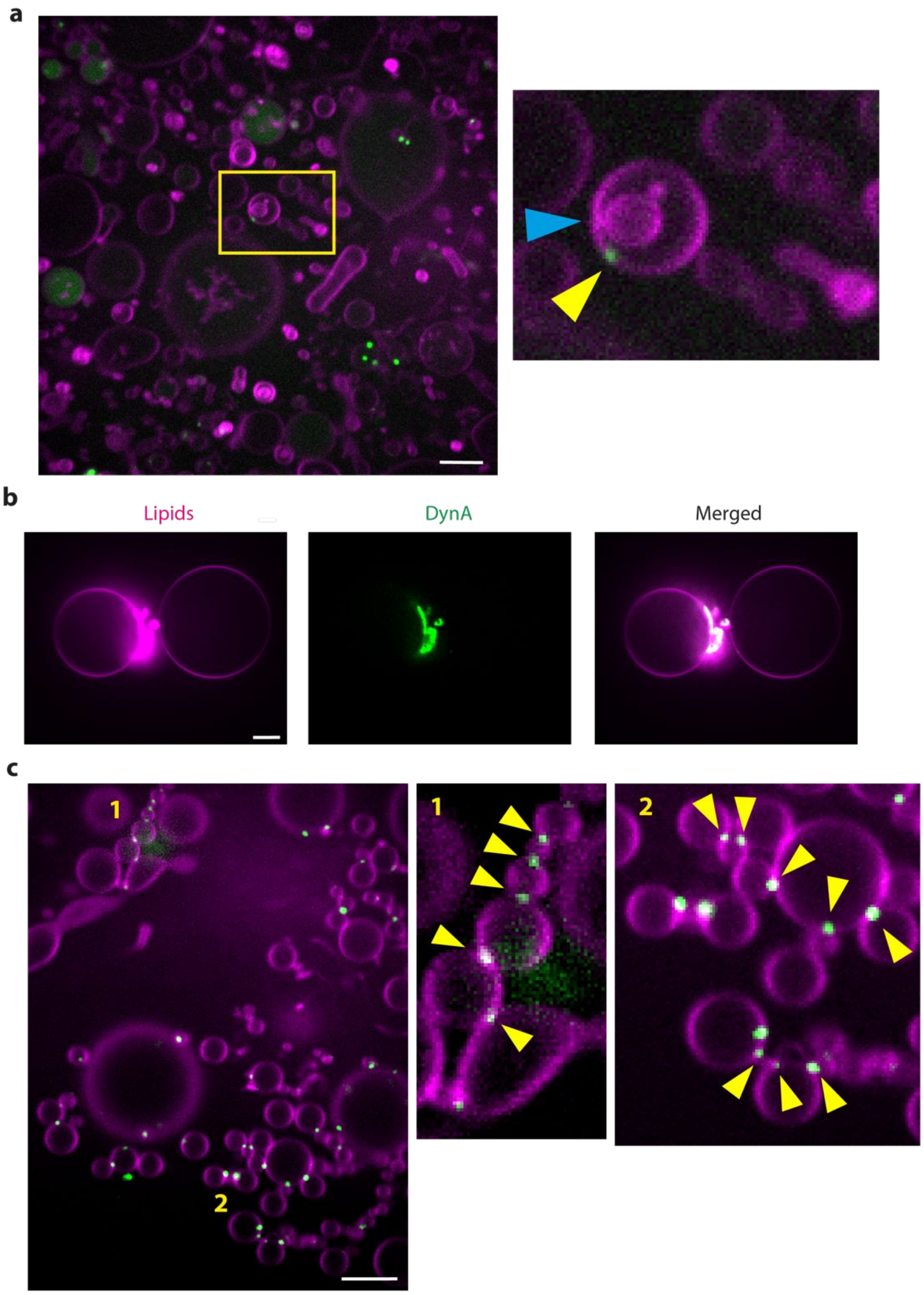

Supplementary Figure 15 - Comparison of different encapsulation methods for DynA. (a): encapsulation of DynA using cDICE and subsequent membrane deformation by changing the osmolarity of the outer buffer. The blue and yellow arrowheads indicate the position of the membrane neck and the DynA cluster, respectively. (b): encapsulation of DynA using the SMS approach but without the addition of chol-oligo and nanostars. (c): encapsulation of DynA using the SMS approach with the addition of chol-oligo and nanostars. Arrowheads indicate the localization of DynA clusters at membrane necks. All scale bars: 10μm

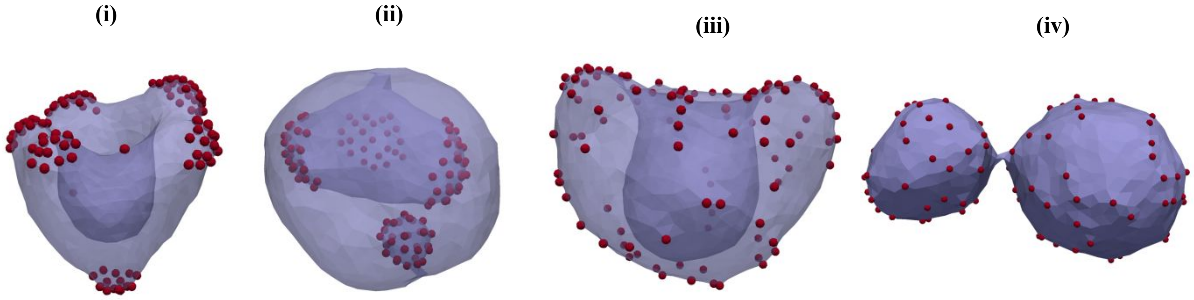

Supplementary Figure 16: Symmetric proteins failed to cluster around the membrane neck. Proteins that do not display asymmetric features, and that therefore do not sense nor generate curvature, fail to cluster at membrane necks in both stomatocyte and dumbbell geometries. Therefore, such a model does not describe the behavior of FTsZ and Dynamin A observed in our experiments. Red spheres represent the proteins.

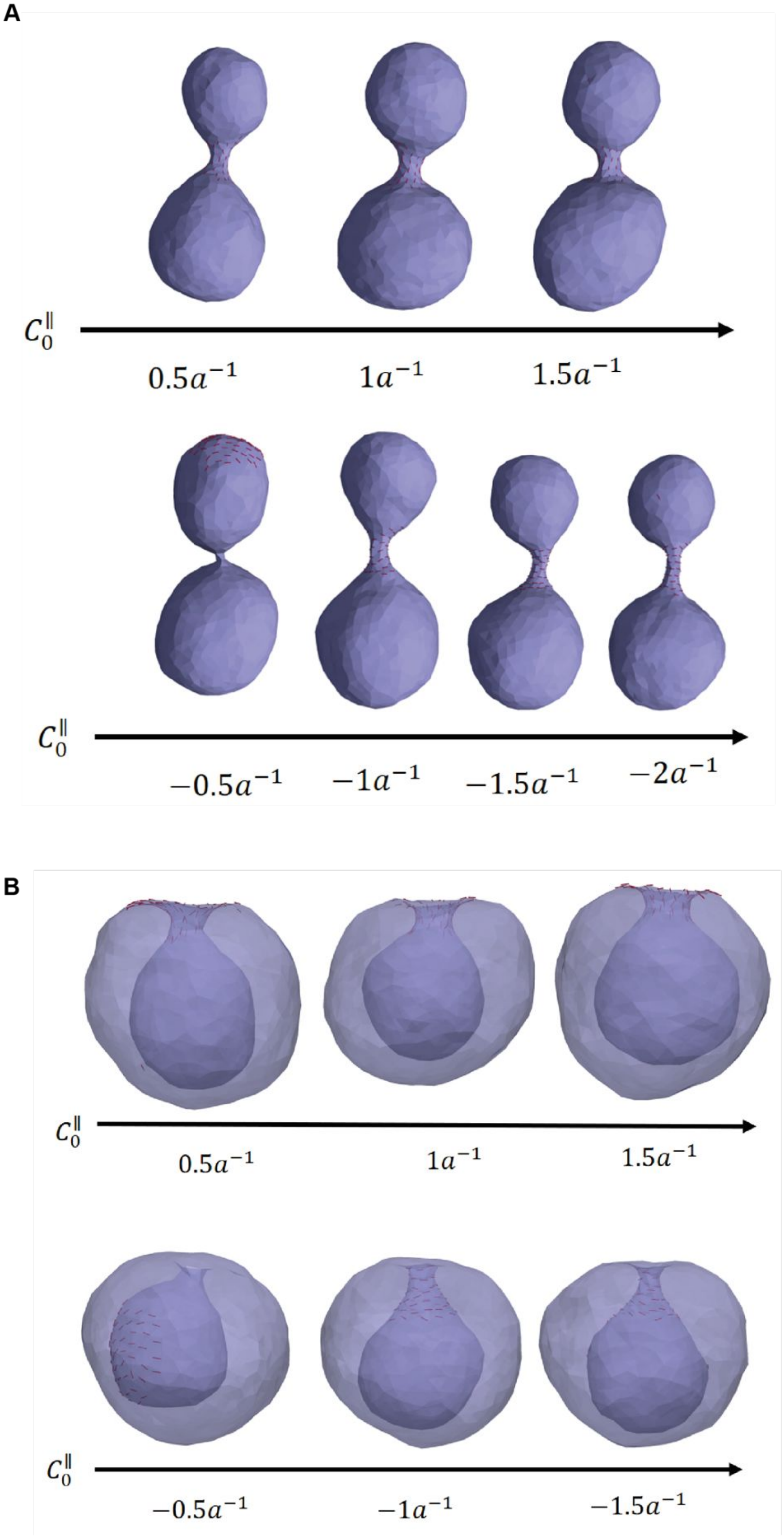

Supplementary Figure 17: Behavior of elongated proteins with different spontaneous curvature around membrane neck. A) Membranes model parameters are $N_{\upsilon} = 1060, \upsilon = 0.7\ c_0 = 0.3[a^{-1}]$;

Protein are bound from inside with 5% coverage. $\kappa_1 = 10kT, \kappa_2 = 0$. **B)** Membranes model parameters are $N_v = 1060, v = 0.45$ $c_0 = 0$; Protein are bound from outside with 5% coverage. $\kappa_1 = 10kT, \kappa_2 = 0$, $\varepsilon_0 = \mu_0 = kT$. Red lines are proteins and the line direction shows the elongation axis. With the exception of one system, all the elongated proteins do cluster in the membrane neck. This results show that only proteins that induce a negative membrane curvature are capable of elongating the neck and adjust the neck to a specific size, while proteins that induce positive membrane curvature only elongating the neck in some rare conditions.

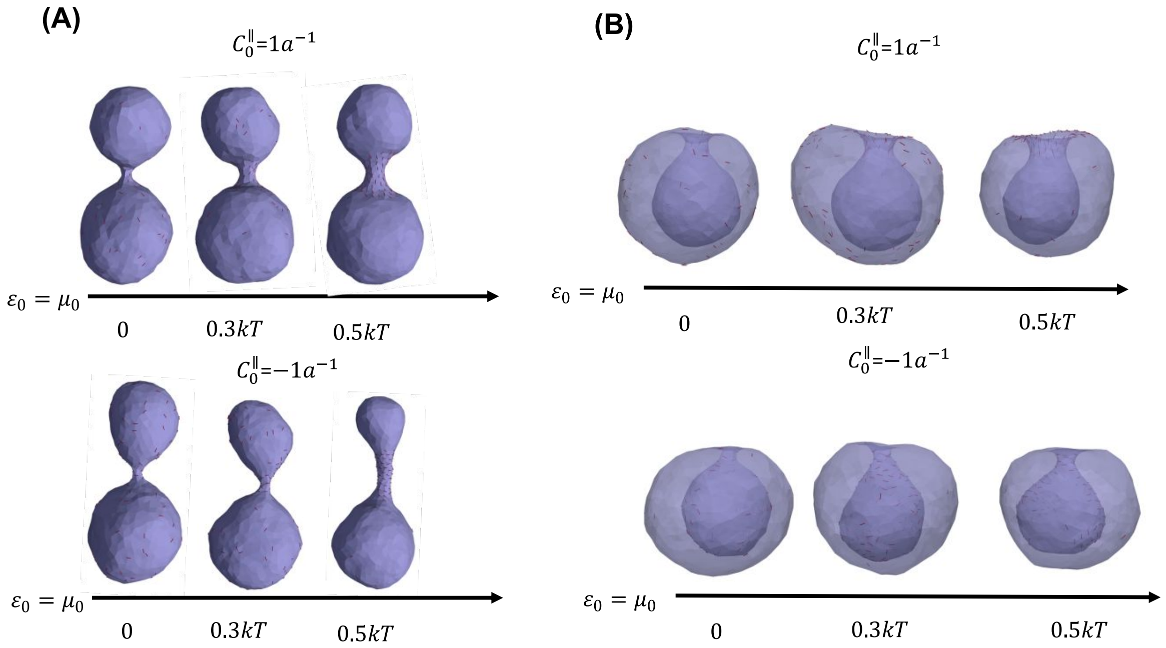

Supplementary Figure 18: Behavior of proteins with different association strength around membrane neck. A) Membranes model parameters are $N_v = 1060, v = 0.7$ $c_0 = 0.3[a^{-1}]$; Protein are bound from inside with 10% coverage. $\kappa_1 = 10kT$,$\kappa_2 = 0$. **B)** Membranes model parameters are $N_v = 1060, v = 0.45$ $c_0 = 0$; Protein are bound from ouside with 10% coverage. $\kappa_1 = 10kT$,$\kappa_2 = 0$. Red lines are proteins and the line direction shows the elongation axis. Protein-protein interaction is required for the proteins to cluster in the membrane neck and the among of clustering is correlated with the interaction strength.

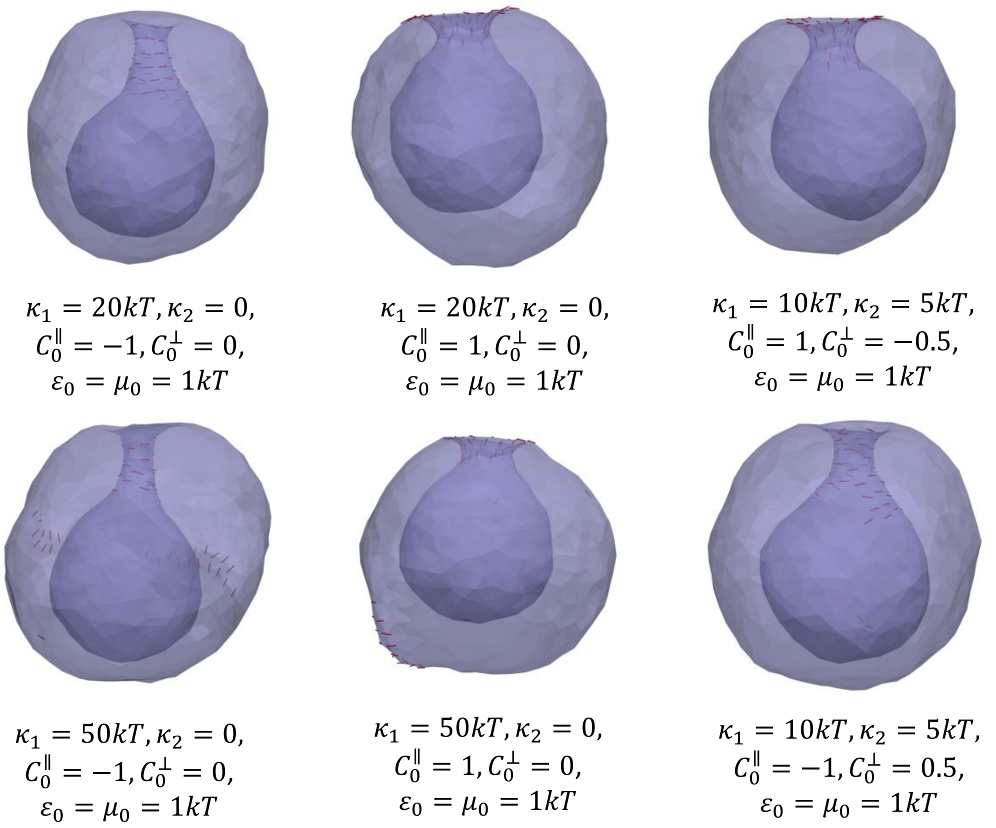

Supplementary Figure 19: Stomatocyte system, robustness of the overall behaviors upon changing protein parameters. 5% protein coverage and proteins are bound from outside and different protein model parameters. Membranes model parameters are $N_v = 1060, v = 0.45\ c_0 = 0.$

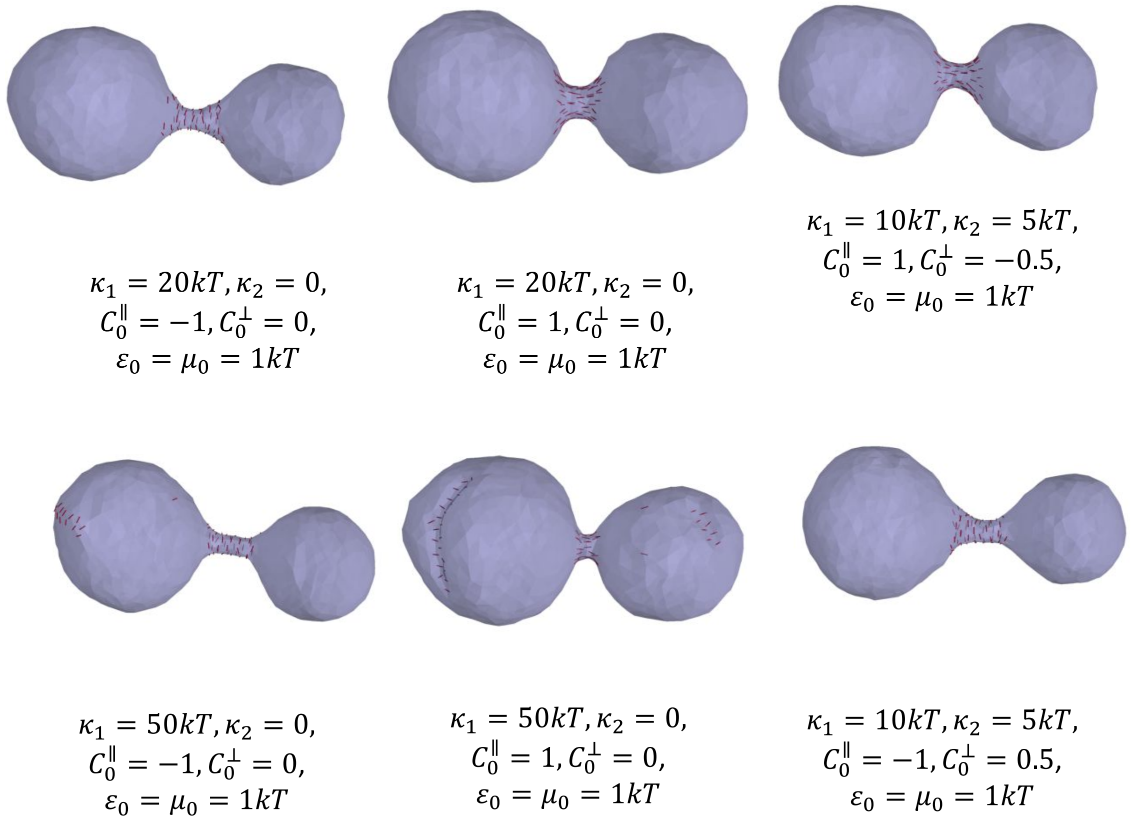

Supplementary Figure 20 - Dumbbell system; robustness of the overall behaviors upon changing protein parameters. 5% protein coverage and proteins are bound from inside and different protein model parameters. Membranes model parameters are $N_v = 1060, v = 0.7\ c_0 = 0.3[a^{-1}]$.

Supplementary Table 1: Average value for PCI, NCI and TC. Error is standard deviation. The first three simulations listed in the table were run for 3 microseconds while the last two were run for 1 microsecond. Note: $PCI > 1$ and $NCI > 1$ indicate association with positive and negative membrane curvature respectively. $TC > 0$ and $TC < 0$ indicate association with positive and negative membrane curvature respectively. See above for the exact definition.

systems	PCI	NCI	TC (nm^{-1})
13-chol-oligo	1.39 ± 0.13	0.46 ± 0.17	0.42 ± 0.11
10-chol-oligo	1.31 ± 0.14	0.58 ± 0.17	0.26 ± 0.11
6-chol-oligo	1.14 ± 0.2	0.5 ± 0.3	0.178 ± 0.11
10-chol	0.82 ± 0.2	1.3 ± 0.3	-0.13 ± 0.1
13 chol-oligo/flat	1.2 ± 0.14	0.85 ± 0.14	0.077 ± 0.075

Supplementary Table 2: comparison between the tube pulling and SMS approaches

	**Tube pulling**	**Synthetic Membrane Shaper**
Curvature sensing by proteins	Yes	Yes
Curvature generation by proteins	Yes	Yes
Force measurement	Yes	No
Control over membrane tension	Yes	No
High throughput	No	Yes
Sample preparation compatible with Cryo-EM	No	Potentially yes
Visualization of membrane scission	Yes	Potentially yes
Protein assembly at toroidal necks	No	Yes
Specialized equipment needed	Yes	No

Supplemetary Table 3: DNA sequences used.

Name	Sequence (5'→3')
NS1	GCTATGGGTGCGTCTGCTCCAGTCATACGTCGTGCGATCTAAAGGCGATGACCAAGGCTATGCAAAACCAGCTACTGCTACGCT
NS2	CTCGCAAAGTAGCATCGACGCATAGCCTTGGTCATCGCCAACACTAGTGGCTCGTCATTCCAAAACCAGCTACTGCTACGCT
NS3	GCTATGGGTGCGTCTGCTCCAGGAATGACGAGCCACTAGTGAACGTGCCACGATCCTATTCGGAAAACCAGCTACTGCTACGCT
NS4	CCGAATAGGATCGTGGCACGAATAGATCGCACGACGTATGACAAAACCAGCTACTGCTACGCT
Chol-oligo#1	CGCACCCATAGCATC-TEG-Cholesterol
Chol-oligo#2	TTTGGGAAGGGGTTCGCAAGTCGCACCCTAAACGA-CholTEG

Supplementary Table 4: inner solutions.

	Stomatocyte	Dumbbell	Stomatocyte + FtsZ:ZipA	Dumbbell + FtsZ:ZipA	Stomatocyte + DynaminA	Dumbbell + DynaminA
Tris pH 7.5	50mM	50mM	50mM	50mM	50mM	50mM
$MgCl_2$	5mM	-	5mM	-	5mM	-
Optiprep	37%	37%	37%	37%	37%	37%
Nanostars	2uM	-	2uM	-	2uM	-
Chol-oligo	4uM	-	4uM	-	4uM	-
FtsZ	-	-	-	100nM	-	-
ZipA-cy5	-	-	-	50nM	-	-
DynaminA-alexa 488	-	-	-	-	-	100nM

Supplementary Table 5: outer solutions.

	Stomatocyte	Dumbbell	Stomatocyte + FtsZ:ZipA	Dumbbell + FtsZ:ZipA	Stomatocyte + DynaminA	Dumbbell + DynaminA
Tris pH 7.5	50mM	50mM	50mM	50mM	50mM	50mM
$MgCl_2$	-	5mM	-	5mM	-	5mM
Glucose	To adjust osmolarity	To adjust osmolarity	To adjust osmolarity	To adjust osmolarity	To adjust osmolarity	To adjust osmolarity
Nanostars	-	250nM	-	250nM	-	250nM
Chol-oligo	-	500nM	-	500nM	-	500nM
FtsZ	-	-	2uM	-	-	-
ZipA-cy5	-	-	1uM	-	-	-
DynaminA-alexa 488	-	-	-	-	200uM	-

Note: Glucose was added stepwise to the outer solution until reaching a value of osmolarity that was 20-40mOsm higher than the osmolarity of the corresponding inner solution.

Supplementary Table 6: lipid mixes composition (expressed in %mol).

	**Stomatocyte**	**Dumbbell**	**Stomatocyte + FtsZ:ZipA**	**Dumbbell + FtsZ:ZipA**	**Stomatocyte + DynaminA**	**Dumbbell + DynaminA**
DOPC	99.9%	99.9%	97.9%	97.9%	94.9%	94.9%
18:1 (Δ9-Cis) PG	-	-	-	-	5%	5%
18:1 DGS-NTA(Ni)	-	-	2%	2%	-	-
18:1 Liss Rhod PE	0.1%	0.1%	0.1%	0.1%	0.1%	0.1%

Movie captions

Movie 1: DTS simulation of shape transformation from a sphere to stomatocyte.

Movie 2: DTS simulation of shape transformation from a sphere to dumbbell.

Movie 3: Movie of a confocal plane across a stomatocyte that was obtained by the SMS. The arrowhead indicates the position of the neck connecting the inner and outer vesicle in the stomatocyte. Lipid fluorescence is shown in yellow. Frame rate was 1 image/sec.

Movie 4: Movie of a confocal plane across a dumbbell that was obtained by the SMS approach but without the addition of chol-oligo/nanostars. The elliptical and fluctuating shape of the lobes indicates low membrane tension. Frame rate was 1 image/sec.

Movie 5: Movie of a confocal plane across a dumbbell that was obtained by the SMS approach with the addition of chol-oligo/nanostars. The constant spherical shape of the lobes indicates high membrane tension. Frame rate was 1 image/sec.

Movie 6: Movie of a confocal plane across a dumbbell that was obtained by the SMS. One lobe is being bleached and lipid fluorescence recovery is shown. Frame rate was 1 image/sec.

Movie 7: Movie of a confocal plane across a stomatocyte that was obtained by the SMS in the presence of Dynamin A, which forms a cluster at the neck of the stomatocyte. Lipid fluorescence is shown in magenta, Dynamin A is in green. Frame rate was 1 image/sec.

Movie 8: Movie of a confocal plane across a stomatocyte that was obtained by the SMS in the presence of FtsZ + ZipA, which led to formation of an array of inward elongated tubes. Lipid fluorescence is shown in magenta, proteins are in cyan. Frame rate was 1 image/sec.

Movie 9: Movie of a confocal plane across a dumbbell that was obtained by the SMS in the presence of FtsZ + ZipA, which led to formation of an elongated neck connecting the two lobes. Lipid fluorescence is shown in magenta, proteins are in cyan. Frame rate was 1 image/sec.

References

1. Fragasso, A. *et al.* Reconstitution of Ultrawide DNA Origami Pores in Liposomes for Transmembrane Transport of Macromolecules. *ACS Nano* **15**, 12768–12779 (2021).
2. A., L., B., M. C., F., K., W., R. & J., E. Absolute and precise measurements of the diffusion of small fluorescent dye molecules across the visible spectrum. in *Poster, 14th International Workshop on Single Molecule Spectroscopy and Ultrasensitive Analysis in Life Sciences* (2008).
